# Chromatin localization of nucleophosmin organizes ribosome biogenesis

**DOI:** 10.1016/j.molcel.2022.10.033

**Published:** 2022-11-23

**Authors:** Ilaria Ugolini, Silvija Bilokapic, Mylene Ferrolino, Josiah Teague, Yinxia Yan, Xuelin Zhou, Ashish Deshmukh, Michael White, Richard W. Kriwacki, Mario Halic

**Affiliations:** 1 Department of Biochemistry, Gene Center, University of Munich (LMU), 81377 Munich, Germany; 2 Department of Structural Biology, St. Jude Children’s Research Hospital, 262 Danny Thomas Place, Memphis, TN 38105, USA; 3 Present address: Faze Medicines, 215 First Street, Suite 440, Cambridge, MA 02142, USA; 4 Lead contact

## Abstract

Ribosome biogenesis takes place in the nucleolus, a nuclear membrane-less organelle. Although well studied, it remains unknown how nascent ribosomal subunits separate from the central chromatin compartment and move to the outer granular component, where maturation occurs. We find that the *Schizosaccharomyces pombe* nucleophosmin-like protein Fkbp39 localizes to rDNA sites encoding the 60S subunit rRNA, and this localization contributes to its specific association with nascent 60S subunits. Fkbp39 dissociates from chromatin to bind nascent 60S subunits, causing the latter to partition away from chromatin and from nascent 40S subunits through liquid-liquid phase separation. *In vivo*, Fkbp39 binding directs the translocation of nascent 60S subunits toward the nucleophosmin-rich granular component. This process increases the efficiency of 60S subunit assembly, facilitating the incorporation of 60S RNA domain III. Thus, chromatin localization determines the specificity of nucleophosmin in sorting nascent ribosomal subunits and coordinates their movement into specialized assembly compartments within the nucleolus.

## In brief

Ugolini et al. use genomics, proteomics, cryo-EM, and biochemistry approaches to investigate the role of fission yeast nucleophosmin-like protein Fkbp39 in ribosome biogenesis. Their findings reveal how the directional movement of ribosome assembly intermediates can be achieved within the nucleolus, a membrane-less organelle organized via liquid-liquid phase separation.

## Graphical abstract

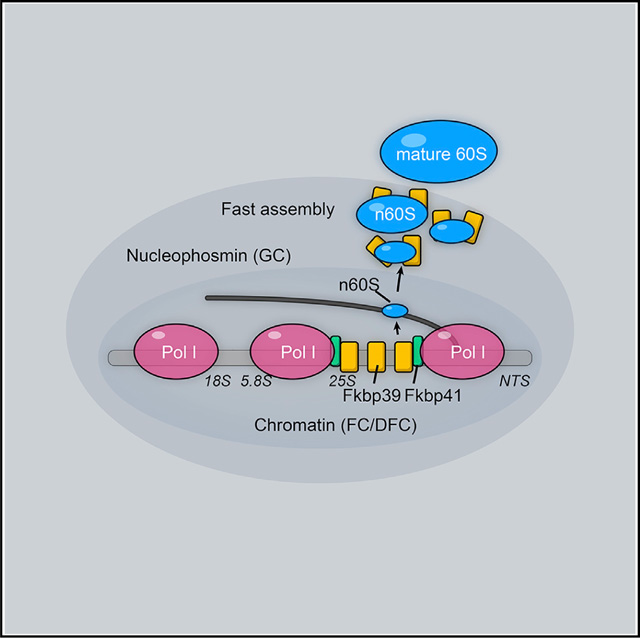

## INTRODUCTION

Eukaryotic ribosome biogenesis is a complex, tightly regulated process that involves many ribosome biogenesis factors and spans different cellular compartments.^1–3^ Ribosome biogenesis starts in the nucleolus, which is organized around the genomic rDNA tandem, each encoding three of the four ribosomal RNAs (rRNAs), 18S, 5.8S, and 25S. The mammalian nucleolus has three compartments: the fibrillar center containing chromatin and rDNA repeats (FC), the dense fibrillar component (DFC) enriched in fibrillarin, and the granular component (GC) enriched in nucleophosmin (NPM1).^4,5^ By contrast, yeast nucleoli seem to have a bipartite organization, with the two central compartments incompletely separated, as the central fibrillar clusters of rDNA repeats show characteristics of both the FC and DFC.^6^ It is well established that the nucleolus is a membrane-less organelle formed through liquid-liquid phase separation (LLPS).^7–14^ Its sub-compartmentalization arises from the immiscibility of different phase-separated fluid regions, comprised of distinct sets of ribosomal components and nucleolar proteins (e.g., fibrillarin and rRNA in the DFC and nucleophosmin, rRNA, and ribosomal proteins in the GC).^9^

During ribosome biogenesis, assembly intermediates move from the internal FC to the outer GC. Indeed, rRNA is transcribed at the interface between the FC and DFC,^15,16^ and assembly starts co-transcriptionally, with early rRNA processing taking place in the DFC and later maturation in the GC.^17^ This vectorial movement is thought to facilitate the assembly line-processing of ribosomes,^13,18^ but it remains unknown how intermediates move from chromatin to the GC and how directionality to the movement is imposed. It is also unclear whether the 40S and 60S ribosomal subunits (40S and 60S hereafter), which mature independently,^19^ partition into different compartments for their assembly. Moreover, although nucleolar sub-compartmentalization has been well described, its requirement for ribosome biogenesis is unclear.

Here, we answer these questions and reveal how the nascent 60S moves from chromatin to the late-assembly compartment in the nucleolus of *Schizosaccharomyces pombe* (*S. pombe*). Using genomics, proteomics, biochemistry, cryo-EM, and fluorescence microscopy imaging approaches, we examined the function of nucleophosmin-like proteins Fkbp39 and Fkbp41. We find that *in vivo* Fkbp39 and Fkbp41 are enriched in genomic rDNA regions corresponding to the 25S rRNA, a component of the 60S. *In vitro*, Fkbp39 binds DNA and nucleosomes but dissociates to bind nascent 60S particles and to partition them from chromatin through LLPS. This partitioning provides directionality for the nascent 60S to move into the nucleophosmin-rich GC, where its biogenesis continues. Furthermore, we show that Fkbp39 is required for efficient rRNA processing and the incorporation of the RNA domain III into the 60S. Our data suggest that chromatin localization of Fkbp39 at 25S rDNA sites determines its specificity for nascent 60S, ultimately resulting in their effective sorting into a different compartment and increasing the efficiency of ribosome assembly.

## RESULTS

### Nucleophosmin associates with chromatin

**Figure 1.**
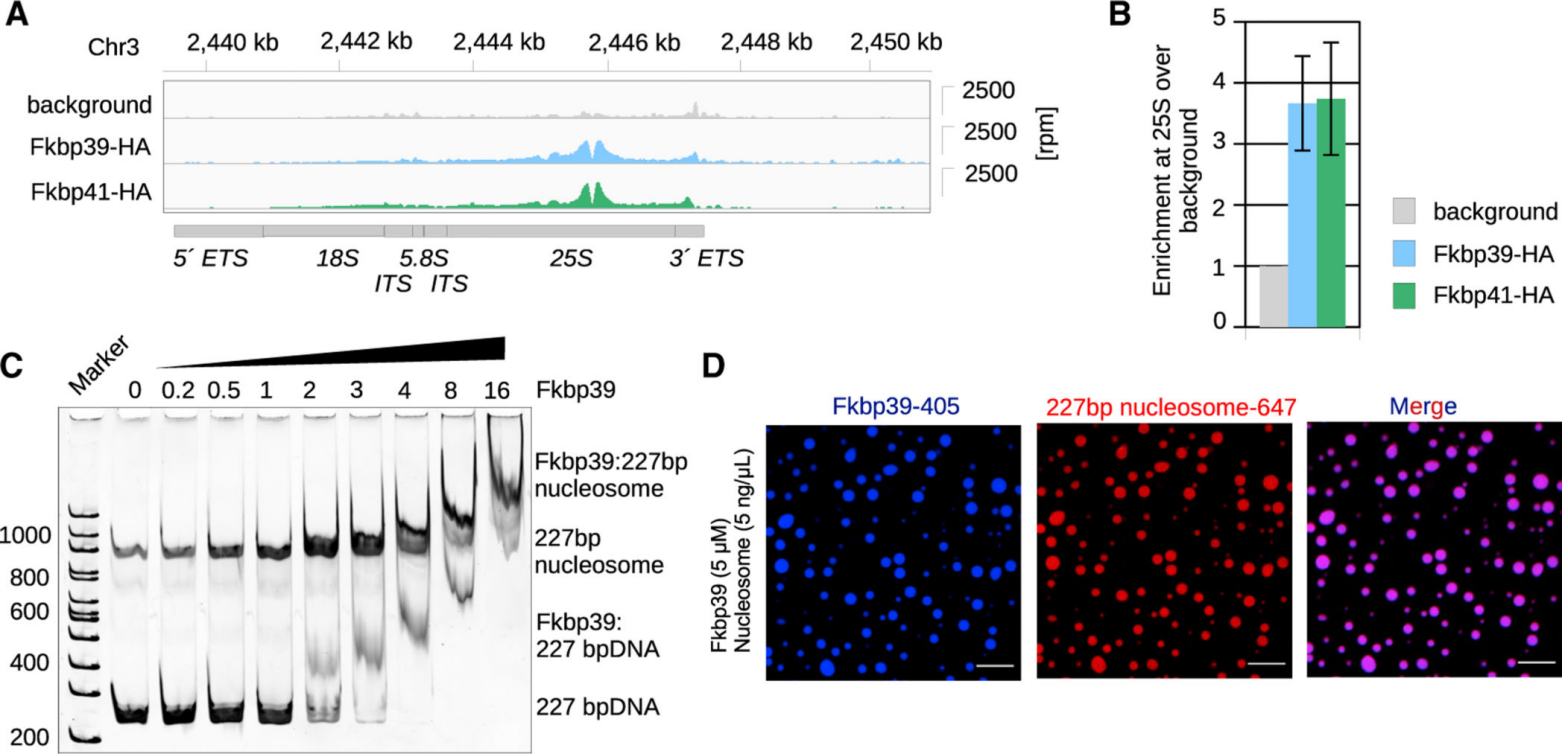
Fkbp39 forms liquid-like condensates with open chromatin (A) ChIP-seq data showing Fkbp39 and Fkbp41 localization at rDNA with a peak over the 25S rDNA. The y axis on the right show reads per million (rpm) normalizedover background regions (see STAR Methods). The coordinates of the genomic location are shown on top, and the rDNA locus organization is depicted below. ETS, external transcribed spacer; ITS, internal transcribed spacer; 18S, small subunit (40S) rRNA; 5.8S and 25S, large subunit (60S) rRNA. (B) Quantification of the reads mapping to the 25S from Fkbp39 and Fkbp41 ChIP-seq data. Reads were normalized to the background. Data shown are mean andSEM of n = 4 independent experiments for Fkbp39 and n = 3 for Fkbp41. (C) *In vitro* binding followed by electrophoresis in 6% PA gel stained with SYBR gold, showing that Fkbp39 binds nucleosomes with 40 bp long linker DNA (227 bp nucleosomes). The relative molar ratio of Fkbp39 to nucleosomes is shown on top (Fkbp39 functional unit is a pentamer). This gel is representative of three independent assays. (D) Confocal fluorescence microscopy images showing Fkbp39:nucleosome condensates. Fkbp39 labeled with CF 405S was mixed with nucleosomes labelled with Alexa 647. Scale bars, 10 μm.

The *S. pombe* genome encodes for two nucleophosmin-like proteins, Fkbp39 (SPBC1347.02) and Fkbp41 (SPAC27F1.06c). Both proteins have an N-terminal nucleophosmin domain, followed by the characteristic intrinsically disordered region, with acidic and basic tracts (Figures S1A and S1B). Despite their overall sequence similarity, we found that Fkbp39 is much more abundant than Fkbp41 in cells and can be found both in insoluble and soluble fractions. By contrast, Fkbp41 is found primarily in the insoluble chromatin fraction (Figure S1C). We then used ChIP-seq to examine the chromatin localization of these two proteins. We found that chromatin-associated Fkbp39 and Fkbp41 predominately localized to the rDNA locus, similar to previous observations on budding yeast and mammalian nucleophosmins,^20–23^ but our ChIP-seq data also revealed a strong enrichment at the 25S rDNA, which had not been described before (Figures 1A, 1B, and S1D). The chromatin localization of Fkbp39 at the 25S rDNA was resistant to RNase A treatment, indicating that the protein binds to DNA or nucleosomes (Figures S1E and S1F).

Nucleophosmin and related proteins were previously shown to bind nucleosomes and act as histone chaperones *in vitro*.^24–27^ To test Fkbp39 and Fkbp41 for such activity, we purified both proteins and observed that they adopt different oligomeric states: Fkbp39 forms a pentamer, whereas Fkbp41 forms a dimer, as determined by mass photometry and size exclusion chromatography (Figures S1G and S1H). We performed *in vitro* binding assays and did not observe the binding of Fkbp41 to DNA or nucleosomes (Figure S1I). On the other hand, Fkbp39 could bind to DNA and nucleosomes with a 40-bp-long linker DNA (Figures 1C, S1I, and S1J) but not to nucleosomes lacking or with short 10 bp linker DNA (Figure S2A). In most of the fission yeast genome, nucleosomes are densely spaced, with linker DNA below 10 bp,^28^ but active rDNA repeats show low nucleosome occupancy, which would allow Fkbp39 binding. For the remainder of this study, we have focused on Fkbp39 and used either DNA or nucleosomes reconstituted with a 227-bp DNA fragment consisting of the 601 positioning sequence and 40 bp overhangs (linker DNA) on both sides as a surrogate for the active rDNA chromatin.

We mutated the active site of the prolyl isomerase domain of Fkbp39 (F301C/W314C/Y337K)^29^ and found that it was impaired in the ability to bind nucleosomes but not DNA (Figure S2B). This suggests that the prolyl isomerase activity contributes to efficient nucleosome binding. In agreement, Fpr4, an Fkbp39 homolog from *Saccharomyces cerevisiae* (*S. cerevisiae*), was shown to isomerize proline residues in the H3 tail.^30^

Human nucleophosmin was previously shown to form liquid condensates.^12,31^ Similarly, we found that Fkbp39, but not Fkbp41, can assemble into homotypic liquid-like condensates *in vitro* (Figure S2C). We assessed the ability of Fkbp39 to form condensates with DNA or chromatin. We found that Fkbp39 can phase separate with nucleosomes and form large liquid-like condensates (Figure 1D). By contrast, Fkbp39 only weakly phase separates with DNA, forming very small condensates at concentrations where homotypic FKBP39 phase separation is favored (Figures S2D–S2F). This supports the role of histones in promoting heterotypic LLPS of Fkbp39 with DNA (Figures 1D and S2E). These observations suggest that Fkbp39 could organize the rDNA-containing chromatin into a phase-separated compartment.

### Fkbp39 interacts with nascent 60S subunits *in vivo*

Next, we pulled down endogenously FLAG-tagged Fkbp39 and determined its interactome by mass spectrometry (Figures S2G–S2H; Table S1; Data S1). We found that Fkbp39 associates with its paralog Fkbp41 and with ribosomal proteins and ribosome biogenesis factors involved in the early maturation of the 60S (Figure S2H; Table S1). These interactions were confirmed by co-immunoprecipitation analyses *in vitro* and *in vivo*, indicating that Fkbp39 interacts with Fkbp41 and nascent 60S (Figures S2I–S2K).

**Figure 2.**
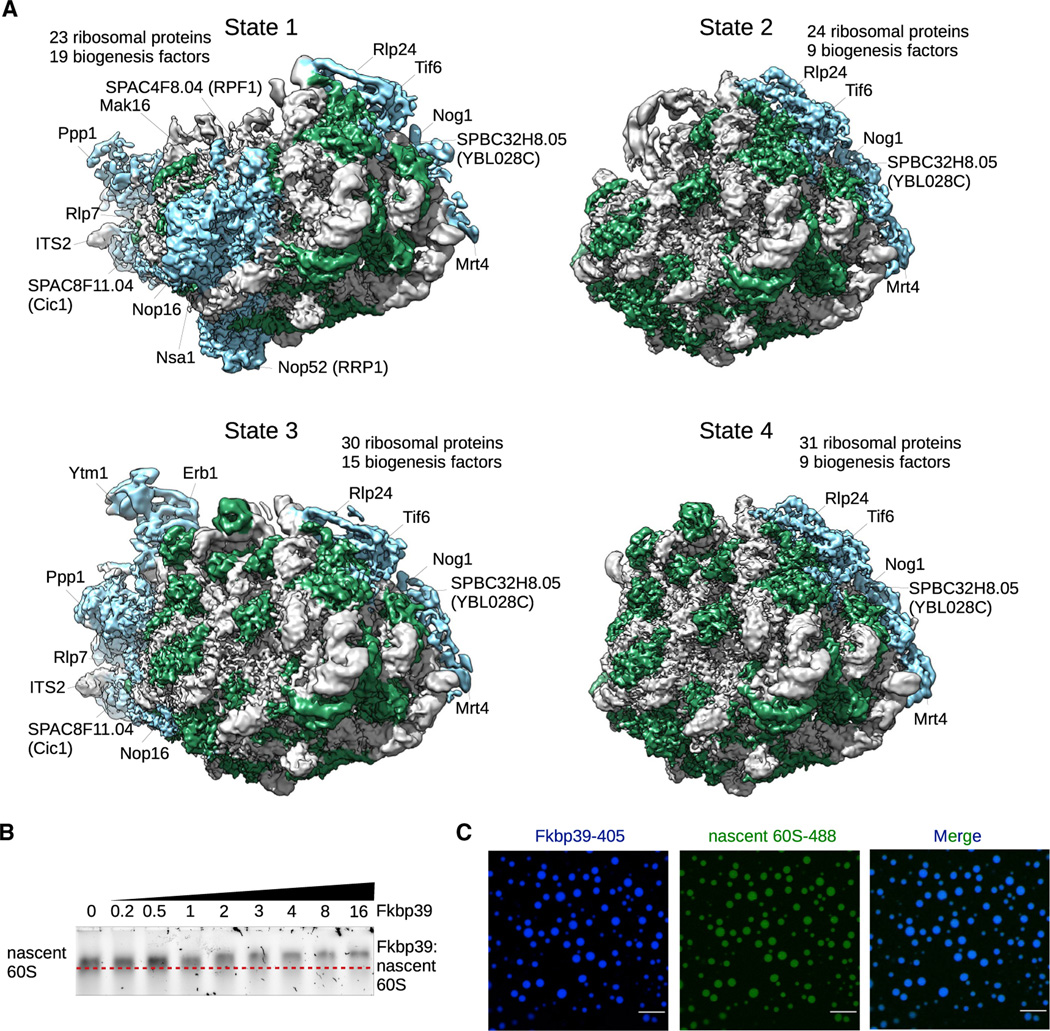
Fkbp39 binds nascent 60S subunits (A) Cryo-EM maps of Fkbp39 pull-down from fission yeast cells showing the 4 major states of nascent 60S, representing biogenesis intermediates found in the nucleophosmin-rich granular compartment (GC) of the nucleolus. rRNA is shown in gray, ribosomal proteins in green, and ribosome biogenesis factors in blue. All maps are filtered to the corresponding local resolution. (B) *In vitro* binding followed by electrophoresis in 0.8% TBE agarose gel stained with SYBR gold, showing that Fkbp39 binds nascent 60S. The relative molar ratio of Fkbp39 to nascent 60S is shown on top (Fkbp39 functional unit is a pentamer). The red dashed line indicates the position of nascent 60S. This gel is representative of three independent assays. (C) Confocal fluorescence microscopy images of Fkbp39:nascent 60S condensates. Fkbp39 labeled with CF 405S was mixed with nascent 60S labeled with Atto 488. Scale bars, 10 μm.

To determine the assembly state(s) of the nascent ribosomes associated with Fkbp39 *in vivo*, we determined the cryo-EM structures of the particles pulled down with Fkbp39. We identified 4 major states of nascent 60S, and each state could be further classified into multiple substates (Figures 2A and S3; Table S1). The nascent 60S associated with Fkbp39 represents early ribosome biogenesis stages as described below. We did not observe clear density for Fkbp39 in the cryo-EM maps, suggesting that it binds nonspecifically to nascent 60S and, therefore, is conformationally disordered. Human nucleophosmin was previously shown to bind to ribosomal proteins or rRNA in a weak and transient manner through multivalent electrostatic interactions^12,31^, the same type of interactions could account for the disorder of Fkbp39 when bound to nascent 60S. Notably, we did not find any nascent 40S particles in the cryo-EM data, which is consistent with the mass spectrometry data and chromatin localization of Fkbp39 on the 25S rDNA (Figure 1A; Table S1).

The structure of state 1 shows an early assembly intermediate with 23 ribosomal proteins and 19 biogenesis factors (Figure 2A; Table S2). This state incorporates the RNA domains I, II, and partially VI, indicating that Fkbp39 is recruited toward the end of transcription, consistent with its chromatin localization at the 25S rDNA region—notably, ITS2 is not cleaved. State 2 has 24 ribosomal proteins but only 9 biogenesis factors, and ITS2 is cleaved and its associated factors are released (Figures 2A and S3D; Table S2). In state 3, RNA domain III is folded and the biogenesis complex Ytm1-Erb1-Ppp1 (Ytm1-Erb1-Nop7 in *S. cerevisiae*) is incorporated^32^ for a total of 30 ribosomal proteins and 15 biogenesis factors (Figure 2A). State 4, the most mature state we detected, has 31 ribosomal proteins, the ITS2 is processed, and its associated factors are released (Figures 2A and S3D).

The assembly intermediates we observe are similar, though not identical, to previously described early 60S structures from budding yeast.^33–35^ States 1 and 2 both resemble *S. cerevisiae* state C,^33^ except that ITS2 is cleaved in state 2, state 3 resembles state E, and state 4 is similar to state E with ITS2 processed (Figure S3D). The variations in composition and conformation among these structures suggest that 60S assembly can occur through multiple pathways, with states 2 and 3 representing intermediates in parallel pathways for RNA domain III incorporation, which can occur before or after ITS2 is processed. Yeast nucleophosmin-like proteins localize mainly in the nucleolus,^36^ and human nucleophosmin partitions 20-fold or more within the GC subcompartment.^13^ Therefore, our data strongly suggest that the 60S subunit intermediates we observe associated with Fkbp39 are undergoing assembly within the nucleolar GC.

### Fkbp39 forms liquid-like condensates with nascent 60S *in vitro*

Similarly to human nucleosphosmin,^12^ Fkbp39 can bind RNA *in vitro* and phase separate with RNA (Figures S4A and S4B). Fkbp39 robustly forms liquid-like condensates with RNA in sharp contrast to condensates with DNA (Figures S4B and S2D). We performed biochemical assays with the nascent 60S pulled down with Fkbp39 and observed that those particles are able to bind additional Fkbp39, suggesting multiple interactions between Fkbp39 and nascent 60S subunits, which is in agreement with our structural observations (Figure 2B). Fkbp39 also forms liquid-like condensates with the isolated nascent 60S (Figure 2C). Co-immunoprecipitation experiments and *in vitro* binding assays show that the prolyl isomerase domain of Fkbp39, but not its activity, is required for its interaction with nascent 60S subunits (Figure S4E).

### Nascent 60S subunits dissociate Fkbp39 from chromatin

**Figure 3.**
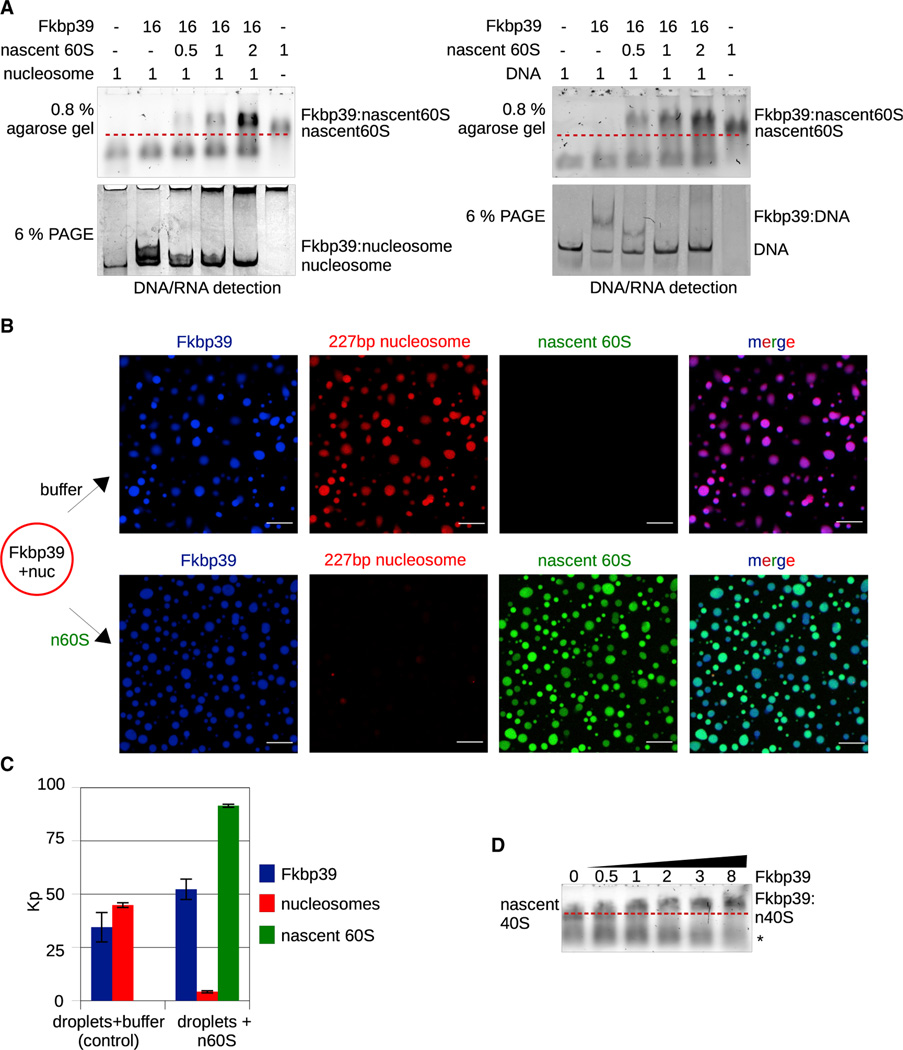
Fkbp39 dissociates from nucleosomes and binds emerging nascent 60S (A) *In vitro* binding followed by electrophoresis showing that Fkbp39 dissociates from nucleosomes or from DNA to bind nascent 60S. Increasing amounts of nascent 60S were added to preformed Fkbp39:nucleosome (left) and Fkbp39:DNA complexes (right). Relative molar ratios of each component are indicated on top (Fkbp39 functional unit is a pentamer). Fkbp39:nucleosome and Fkbp39:DNA complexes are visualized on 6% native PA gel, Fkbp39:n60S complexes on 0.8% TBE agarose gel. Red dash lines indicate the position of the nascent 60S. The gels shown are representative experiments of three independent assays. (B) Confocal fluorescence microscopy images showing that nascent 60S displace nucleosomes from Fkbp39:nucleosome condensates. Fkbp39 is labeled withCF 405S, nucleosomes with Alexa 647, and nascent 60S with Atto 488. Images are representative of one of two independent experiments. Scale bars, 10 μm. (C) Partition coefficient (Kp) from experiments shown in (B). Kp values for each component were determined from more than 300 condensates. For nascent 60S and nucleosomes, Kp values were normalized to their mass (described in detail in STAR Methods). Graph shows mean and SD. (D) *In vitro* binding followed by electrophoresis in 0.8% TBE agarose gel stained with SYBR gold, showing Fkbp39 binding to nascent 40S. The relative molar ratio of Fkbp39 to nascent subunits is indicated (Fkbp39 functional unit is a pentamer). The red dashed line indicates the position of nascent ribosome subunits. The asterisk marks a non-specific band. This gel is the representative experiment of three independent assays.

Our data suggest that the specific binding to nascent 60S *in vivo* could be due to Fkbp39 localization at 25S rDNA sites enabling co-transcriptional partitioning of the protein to nascent particles. To test this hypothesis, we pre-assembled either Fkbp39-nucleosome or Fkbp39-DNA complexes, then added increasing amounts of nascent 60S, mimicking their emergence during the early steps of biogenesis. We observed the formation of Fkbp39-nascent 60S complexes with concomitant disassembly of Fkbp39-nucleosome and Fkbp39-DNA complexes (Figure 3A). Moreover, nascent 60S could enter preformed Fkbp39-nucleosome condensates and displace the nucleosomes (Figures 3B and 3C). Our data suggest that Fkbp39 could dissociate from chromatin to bind emerging nascent 60S.

To determine if there is directionality in this “switch” mediated by Fkbp39, we tested whether DNA or nucleosomes could displace Fkbp39 associated with nascent 60S. Fkbp39 was pre-bound to the nascent 60S, and we then added increasing amounts of nucleosomes or DNA, but neither could displace Fkbp39 from nascent 60S (Figure S4F). Similarly, nucleosomes could not displace nascent 60S in preformed Fkbp39-nascent 60S condensates and partitioned very little into them (Figures S4G and S4H).

### Specific association of Fkbp39 with nascent 60S is imparted by chromatin localization

As shown above, Fkbp39 associates specifically with nascent 60S but not with nascent 40S *in vivo*. We thus verified whether Fkbp39 could associate with nascent 40S *in vitro*. We isolated nascent 40S (also known as small subunit processome) by pulling down fibrillarin (Nop1)-associated particles (Figure S4I; Table S3; Data S1) and observed that Fkbp39 could associate with nascent 40S with an apparent affinity comparable to with nascent 60S (Figures 3D and S4J). We also tested whether DNA-bound Fkbp39 could bind nascent 40S *in vitro*. We observed that Fkbp39 could dissociate from DNA to bind nascent 40S, but DNA could not displace Fkbp39 from preformed complexes with nascent 40S (Figure S4K).

Thus, *in vitro*, Fkbp39 can associate with either nascent 40S or nascent 60S in a similar manner. These observations argue against Fkbp39 binding to specific ribosomal proteins or biogenesis factors present in the nascent 60S. Instead, they suggest that the specificity of Fkbp39 for nascent 60S *in vivo* is imparted by the protein’s localization on 25S rDNA chromatin. This idea is supported by our cryo-EM and biochemistry data, which indicated multiple weak and dynamic interactions between Fkbp39 and nascent 60S.

### Fkbp39 separates nascent ribosomes from chromatin in cells

Our *in vitro* data are consistent with the general framework of cotranscriptional ribosome assembly: transcription occurs in the chromatin environment within the nucleolus, where the first steps of ribosome biogenesis take place. Importantly, our data support a model with several testable features. In our model, Fkbp39 is initially bound to active rDNA repeats, associates with emerging nascent 60S, and separates that particle from chromatin and into a new compartment where ribosome biogenesis proceeds. We propose that there is a constant flux of Fkbp39 molecules through these sequential steps.

**Figure 4.**
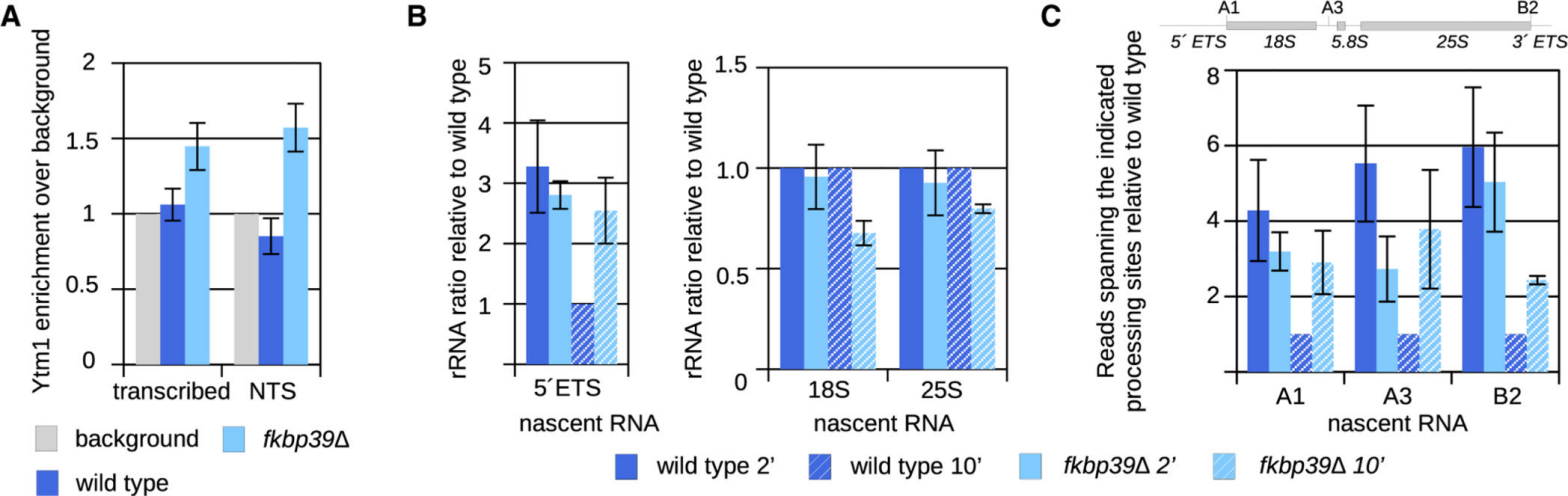
Nascent 60S are retained on chromatin in *fkbp39Δ* cells (A) Quantification of the reads mapping over to rDNA locus from Ytm1 ChIP-seq data from wild-type and *fkbp39Δ* cells (Figure S5A). Reads were normalized to the background. “Transcribed” represents the entire rDNA transcriptional unit. Data are shown as mean and SEM from six independent experiments. (B) Quantification of nascent RNA sequencing from wild-type and *fkbp39Δ* cells. Sense reads mapping to the 5′ ETS, 18S, and 25S RNA was quantified and normalized to the amount of total reads. Wild-type values were set to 1. Data are shown as mean and SEM from three independent experiments. (C) Quantification of nascent RNA sequencing spanning rRNA processing sites. A schematic representation of the processing sites is above the graph. Sense reads spanning the indicated processing sites were quantified and normalized to the amount of rRNA sense transcripts, and the value for wild-type strain at the 10-min time point was set to 1. Data are shown as mean and SEM from three independent experiments.

To test this hypothesis *in vivo*, we performed ChIP-seq analyses to determine the chromatin localization of ribosome biogenesis factor Ytm1, a component of the nascent 60S (Figure 2A). In wild-type cells, Ytm1 is not detectable on chromatin, in agreement with our *in vitro* data showing that Fkbp39 separates ribosome biogenesis intermediates from chromatin (Figures 4A, S5A, and S5B). By contrast, in *fkbp39Δ* cells, we detected Ytm1 on chromatin, primarily at the 3′ end of the rDNA repeats, specifically enriched at the 3′ ETS and the non-transcribed spacer (NTS) DNA sites (Figures 4A, S5A, and S5B). Consistent with chromatin localization, the mass spectrometry analysis of Ytm1 pull-downs from *fkbp39Δ* cells reveals that nascent 60S interacts with the histone chaperone complex FACT (Figure S5C).

These data show that nucleophosmin proteins are required for proper compartmentalization of ribosome biogenesis within the nucleolus. In the absence of Fkbp39, Ytm1-containing nascent 60S remain in proximity to chromatin and accumulate toward the 3′ end of the rDNA transcriptional unit and in intergenic regions. These observations support our model that Fkbp39 partitions nascent 60S away from chromatin and into a distinct environment.

### Compartmentalization of nascent 60S is required for biogenesis

We next asked whether the compartmentalization of nascent 60S by Fkbp39 is required for ribosome biogenesis. We analyzed rRNAs in wild-type and *fkbp39Δ* cells by RNA sequencing (RNA-seq) and found reduced levels of 18S and 25S rRNA and accumulation of 5′ ETS RNA and unprocessed rRNA in the mutant compared with wild-type cells (Figures S5D and S5E).

To determine if Fkbp39 is required for transcription or RNA processing, we performed nascent RNA-seq using 4-thiouracil labeling. Immediately after labeling, the levels of nascent rRNA in wild-type or *fkbp39Δ* cells were comparable (Figures 4B, S5F, and S5G), indicating that Fkbp39 is not required for rRNA transcription. However, at a later time point (10 min after labeling), we observed the accumulation of unprocessed rRNA at nearly all processing sites and of 5′ ETS RNA, as well as a reduction in 18S and 25S rRNA (Figures 4B, 4C, and S5H–S5J). These data indicate defects in rRNA processing in *fkbp39Δ* cells.

We next analyzed ribosomes by sucrose gradient centrifugation. Compared with wild-type cells, we observed the accumulation of RNA in the void (nonribosomal) and 40S subunit fractions in *fkbp39Δ* cells, accompanied by a decrease in the 60S/80S and polysome fractions (Figures S5K–S5M). These findings indicate that in *fkbp39Δ* cells, 40S subunits are in excess relative to 60S subunits, thus limiting the assembly of 80S ribosomes. Although this observation might appear in conflict with the defect in 18S processing detected in *fkbp39Δ* cells, this is not the case since the excess of 40S relative to 60S does not exclude biogenesis defects in the former. Together, these findings merely show that defects in biogenesis are more severe for nascent 60S than for nascent 40S.

The reduced amount of 80S ribosomes in *fkbp39Δ* cells affects cell growth, as evidenced by the slower growth of *fkbp39Δ* cells compared with the wild type (Figure S5N). When wild-type and *fkbp39Δ* cells were co-cultured, the population was quickly overtaken by the wild-type strain, leading to the loss of *fkbp39Δ* cells (Figure S5O).

To understand the molecular basis of the defects in 60S biogenesis caused by the absence of Fkbp39, we tagged endogenous Ytm1 and analyzed whole-cell lysates or isolated (Ytm1 pull-down) nascent 60S fractions from wild-type and *fkbp39Δ* cells. The levels of total Ytm1 were slightly reduced in *fkbp39Δ* cells compared with the wild type, but interactions with nascent 60S were comparable in mutant and wild-type cells, as shown by analyses of the pulled-down samples (Figures S6A and S6B). These data indicate that Fkbp39 is not required for Ytm1 binding to nascent 60S.

**Figure 5.**
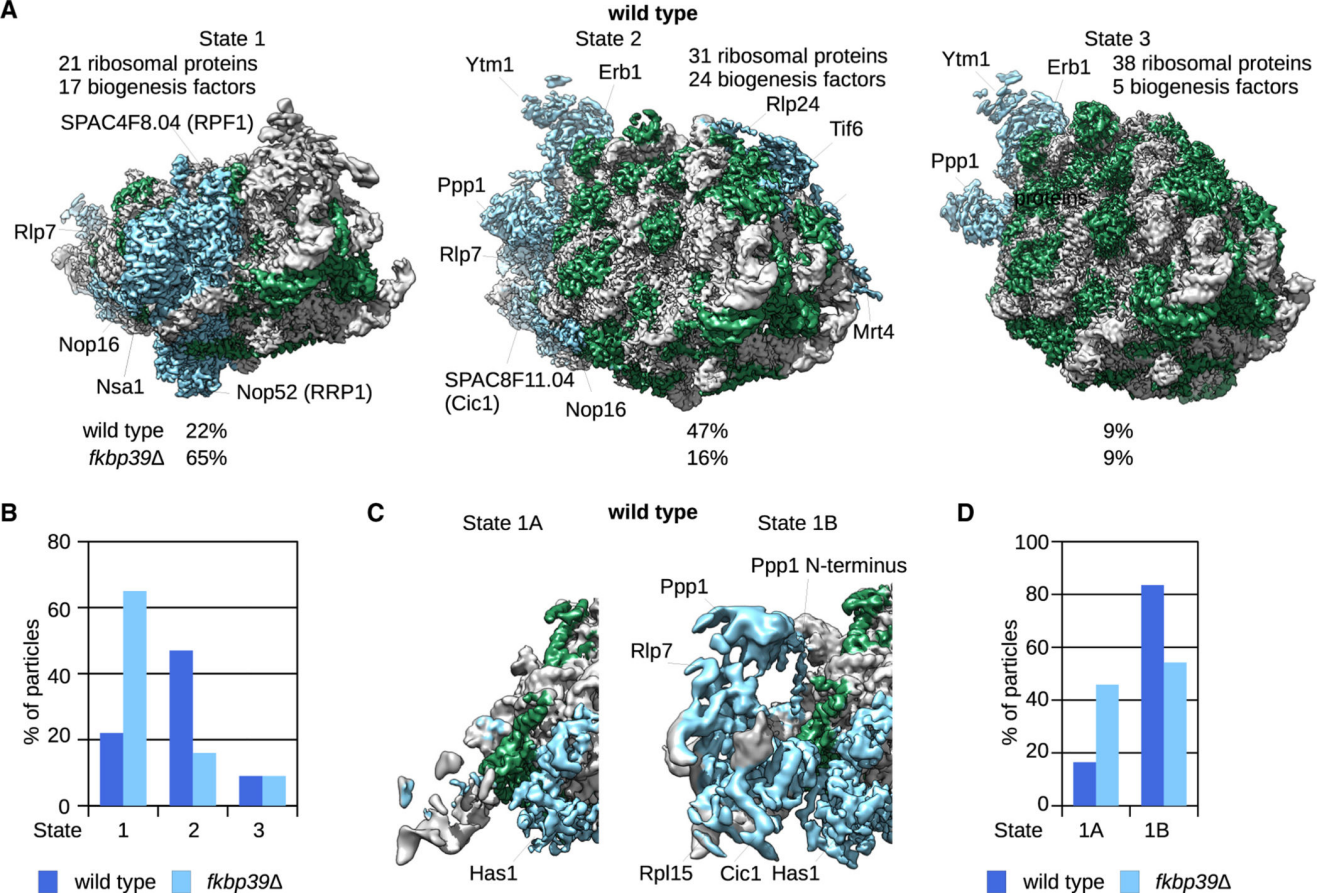
Compartmentalization of nascent 60S subunits promotes the progression of the assembly pathway (A) Cryo-EM maps showing the 3 major states of nascent 60S associated with the biogenesis factor Ytm1 in wild-type cells. rRNA is shown in gray, ribosomalproteins in green, and ribosome biogenesis factors in blue. All maps are filtered to the corresponding local resolution. B) Quantification of particles populating the 3 states shown in panel A in wild-type and *fkbp39Δ* cells. C) Structural detail showing differences between state 1A and 1B. Ppp1 and Rlp7 are associated with nascent 60S subunits in 1B but not in 1A. (D) Quantification of particles in states 1A and 1B in wild-type and *fkbp39Δ* cells.

We analyzed the isolated Ytm1-containing nascent 60S by cryo-EM, which revealed 3 major states in both wild-type and *fkbp39Δ* cells—each state can be further classified into multiple substates (Figures 5A and S6C–S6H; Table S4). States 1 and 2 are similar to the nascent 60S particles pulled down with Fkbp39 (Figure 2A) and with *S. cerevisiae* states B/C and E, respectively^33^ (Figure S3D), and correspond to early or intermediate conformations in the assembly. Among other differences, the RNA domain III is flexible in state 1 but stably incorporated in state 2. State 3 represents a more mature particle, with the 60S already assembled but with the biogenesis complex Ytm1-Erb1-Ppp1 still present, and there was no similar state in the Fkbp39-associated material, indicating that state 3 is a post-nucleolar particle.

Notably, these 3 states look nearly identical in wild-type and mutant cells (Figures 5A, S6I, and S6J), but their distribution differed markedly. In wild-type cells, ~20% of particles are in state 1 and 50% in state 2, and in *fkbp39Δ* cells, the populations are ~65% and 15%, respectively (Figure 5B). The accumulation of state 1 particles in *fkbp39Δ* cells indicates that Fkbp39 is required for a step in 60S biogenesis along the transition from state 1 to state 2.

To gain deeper insight into those biogenesis defects, we characterized state 1 in more detail. In the wild-type dataset, the majority of particles (~85%) had density for Ppp1 and associated proteins (state 1B) (Figures 5C, 5D, S7A, and S7B), whereas in the *fkbp39Δ* dataset, only ~55% of particles in state 1 exhibited density for those factors. Furthermore, in the wild-type dataset, state 1B showed the N-terminal helices of Ppp1 stably incorporated into the nascent 60S subunit, but those helices were less defined in the nascent subunit isolated from *fkbp39Δ* cells (Figures 5C and S7B). These data suggest defective ITS2 processing, and in agreement, we observe the accumulation of ITS2 in Ytm1-associated particles in fkbp39D cells (Figure S7C). Our data indicate that Fkbp39 is required for the stable incorporation of Ppp1 and of the Ytm1-Erb1-Ppp1 complex into nascent 60S particles, which is essential for ITS2 processing and the assembly of the RNA domain III. In support of this conclusion, we observe reduced levels of several ribosomal proteins in Ytm1-containing particles in *fkbp39Δ* cells (Figure S7D). We also observe that in *fkbp39Δ* cells nascent 60S bind eIF3 complex, indicating that a subset of nascent 60S might be involved in translation (Figure S7D).

### Fkbp41 recruits Fkbp39 to 25S rDNA

**Figure 6.**
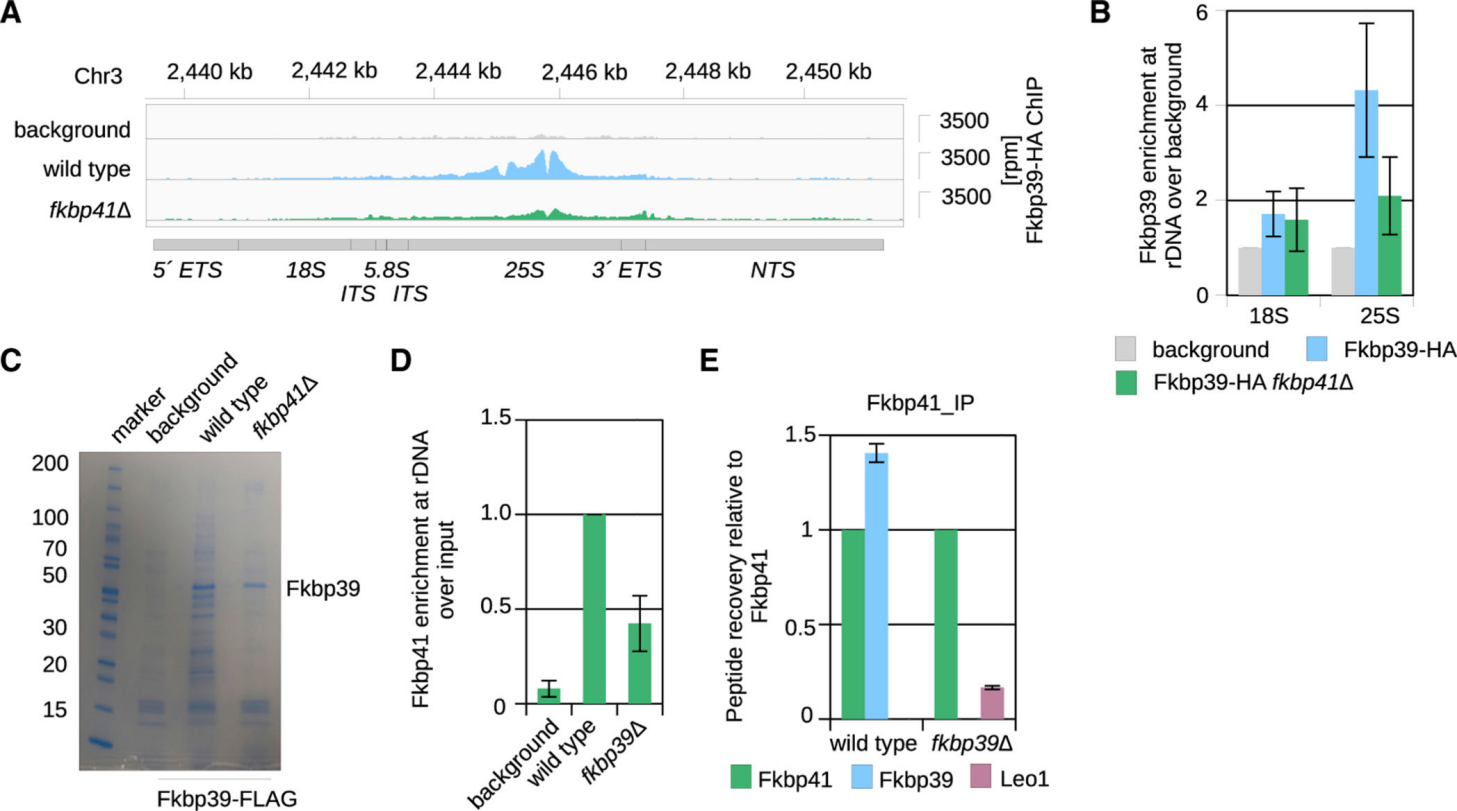
Fkbp41 recruits Fkbp39 to 25S rDNA (A) ChIP-seq data showing Fkbp39 localization at rDNA in wild-type and *fkbp41Δ* cells. The y axis on the right shows reads per million (rpm) normalized over background regions (see STAR Methods). The coordinates of the genomic location are shown on top, the rDNA locus organization is depicted below as in Figure 1A. (B) Quantification of the reads mapping to the 18S and 25S rDNA regions from Fkbp39 ChIP-seq data in wild-type and *fkbp41Δ* cells. Reads were normalized to the background. Data shown are mean and SEM from two independent experiments. (C) Representative SDS-PAGE with Coomassie blue staining showing the Fkbp39-FLAG pull-down samples from wild-type and *fkbp41Δ* cells that were analyzed by mass spectrometry. (D) ChIP-qPCR experiment showing Fkbp41 enrichment at rDNA in indicated strains. Fkbp41 enrichment at rDNA was calculated over input, and the enrichmentin the wild-type background was set to 1. Data shown are mean and SEM from three independent experiments. (E) Mass spectrometry data of samples immunoprecipitated with Fkbp41 from wild-type and *fkbp39Δ* cells, showing the interaction with the transcription elongation factor Leo1. Peptide counts of the indicated proteins were normalized to total spectral counts and plotted relative to Fkbp41 peptide counts. Data shown are mean and SEM from 2 independent experiments. A complete list of the mass spectrometry results is in supplemental information.

Our *in vitro* nucleosome-binding assays (Figures S1I and S1J) indicated that Fkbp39 associates specifically with chromatin regions with an open structure, which is found at active rDNA repeats. However, it remains unclear how Fkbp39 is enriched specifically at 25S rDNA, as seen in our ChIP-seq data (Figure 1A). Since Fkbp41 also localizes to rDNA (Figure 1A) and interacts with Fkbp39 (Figures S2I and S2J), we asked whether Fkbp41 contributes to Fkbp39 recruitment by performing ChIP-seq analyses of Fkbp39 in *fkbp41Δ* cells. Although the overall Fkbp39 protein levels are slightly reduced in *fkbp41Δ* total lysates (Figure S7E), there was a marked reduction of Fkbp39 localization specifically at 25S rDNA in those cells compared with wildtype (Figures 6A, 6B, and S7F). These data suggest that Fkbp41 facilitates Fkbp39 recruitment to the 25S rDNA.

The reduced Fkbp39 levels at the 25S rDNA in *fkbp41Δ* cells allowed us to directly assess whether the chromatin localization of Fkbp39 is necessary for its interaction with nascent 60S subunits. We pulled down tagged Fkbp39 from cell lysates and by SDS-PAGE and mass spectrometry analyses, we observed a substantial reduction in the amount of nascent 60S associated with Fkbp39 in *fkbp41Δ* cells compared with wild-type cells (Figure 6C; Table S5). This is not because Fkbp41 promotes Fkbp39 interaction with nascent 60S as observed in our *in vitro* binding assays (Figure S7G). Together, the ChIP-seq and pull-down data show that the localization of Fkbp39 at 25S rDNA chromatin is required for its loading onto nascent 60S subunits *in vivo*.

Although Fkbp41 localizes to rDNA repeats *in vivo*, the protein does not bind DNA or nucleosomes *in vitro* (Figures 1A and S1I). By ChIP-qPCR assays, we found that Fkbp41 still localizes at rDNA in *fkbp39Δ* cells, though at a lower level than in wild-type cells (Figure 6D). We tagged endogenous Fkbp41 and performed pull-downs from wild-type and *fkbp39Δ* cells, followed by a mass spectrometry analysis to determine its interactome—several interactions were confirmed by co-immunoprecipitation. We found that Fkbp41 interacts with nascent 60S subunits, including Ytm1-containing particles *in vivo* (Table S6)—we confirmed that interaction *in vitro* (Figure S7H). Fkbp41 also interacts with components from the transcription machinery, specifically Leo1, a subunit from the Paf1 complex, which promotes RNA Polymerase I transcription elongation (Figures 6E and S7I).^37^ No interactions between Fkbp39 and transcription elongation factors were detected by mass spectrometry (Table S5). Overall, our data suggest that Fkbp41 is recruited to the 25S rDNA site by interaction with the transcription machinery, where it promotes specific Fkbp39 localization.

## DISCUSSION

Our *in vivo* data show that the two *S. pombe* nucleophosmin proteins Fkbp39 and Fkbp41 localize to chromatin at active rDNA repeats, mostly at 25S rDNA sites, and interact with nascent 60S. Our *in vitro* and *in vivo* analyses suggest that Fkbp39 dissociates from chromatin as the nascent 60S emerge, binding to those subunits, and partitioning them away from the chromatin environment. We propose that this re-partitioning of nascent 60S leads to the formation of a distinct compartment that is enriched in Fkbp39, namely GC,^5,9,13^ where ribosome biogenesis proceeds.

**Figure 7.**
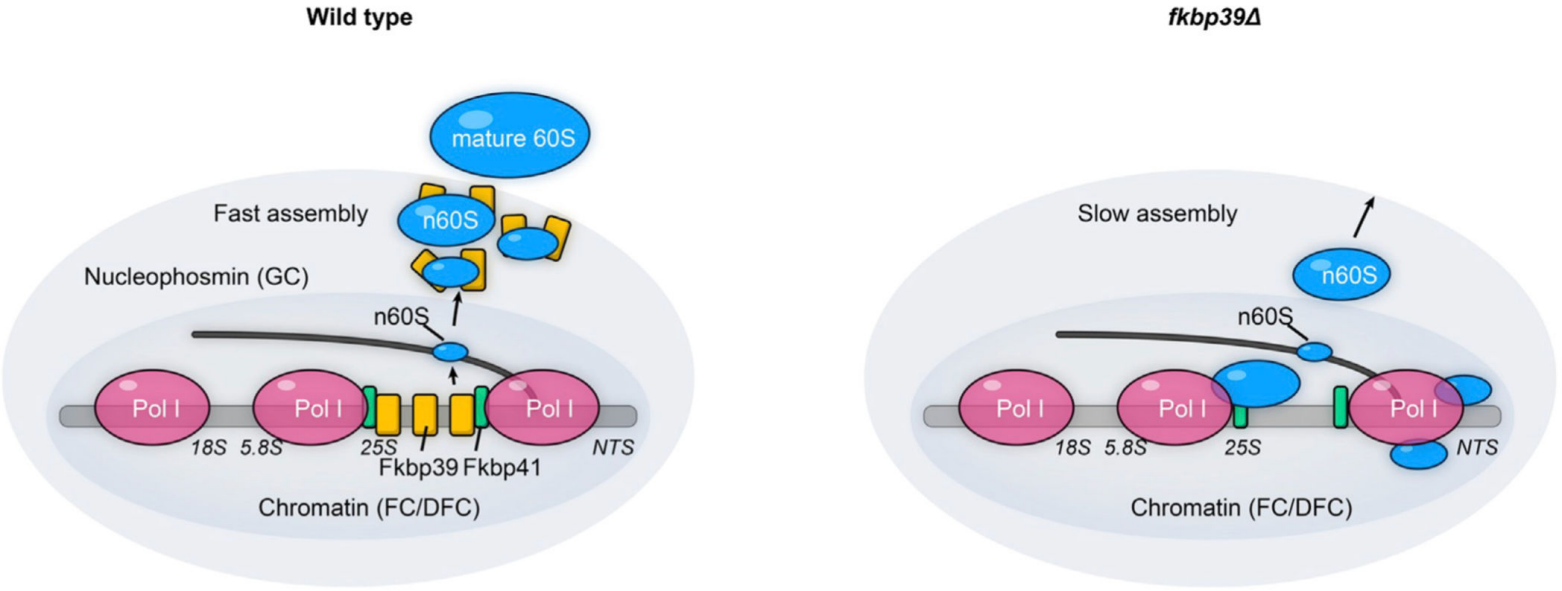
Nucleophosmin separates nascent 60S from chromatin and organizes them into a specific compartment where maturation proceeds Cartoon schematic showing that nucleophosmin protein Fkbp41 (green rectangle) is recruited by the transcription machinery to the rDNA locus, specifically over the 25S region, where it in turn recruits Fkbp39 (yellow rectangle). As nascent 60S emerge cotranscriptionally, Fkbp39 and Fkbp41 dissociate from chromatin to bind them, causing their partitioning away from chromatin through liquid-liquid phase separation. This partitioning process leads to the formation of a distinct compartment enriched in nucleophosmin, namely the granular component (GC), where ribosome biogenesis proceeds. In the absence of Fkbp39, the nascent 60S subunits are retained on chromatin, in particular, toward the end of the transcriptional unit, which results in delayed assembly of 60S subunits.

Our data also suggest that the segregation of 60S away from chromatin likely occurs through LLPS, a process we could recapitulate *in vitro*, showing nascent 60S can replace nucleosomes and DNA in preformed condensates with Fkbp39. Notably, the directionality of this process assures that nascent 60S subunits move from the inner chromatin environment toward the outer GC (Figure 7). Our *in vivo* observations support this model: in the absence of Fkbp39, nascent 60S subunits accumulate on chromatin.

This re-partitioning process segregates the biogenesis of 60S away from chromatin and, possibly, from 40S assembly. The latter is in agreement with recent findings showing that biogenesis factors specific for the assembly of 40S or 60S are located in distinct nucleolar layers in budding yeast^38^ and that nascent 40S and 60S have distinct localization patterns in *Chlamydomonas reinhardtii*.^39^

We showed that Fkbp39 interacts primarily with nascent 60S *in vivo*, though *in vitro*, it binds equally well to either nascent 60S or nascent 40S. These observations show that Fkbp39 specificity for nascent 60S subunits *in vivo* is not due to intrinsic binding affinity. Instead, our data suggest that Fkbp39 localization specifically at 25S rDNA sites positions this protein for association with nascent 60S (Figure 7). We also find that Fkbp41 promotes the specific enrichment of Fkbp39 at the 25S rDNA region, but an additional investigation will be required to fully understand this mechanism.

Although the sub-compartmentalization of the nucleolus has been well described, its role remains elusive. Nascent RNA-seq from *fkbp39Δ* cells revealed a delay in rRNA processing, with the accumulation of unprocessed rRNA at almost all processing sites. The defects in 60S biogenesis in *fkbp39Δ* cells occur at multiple steps and indicate an overall delay in the assembly process, in agreement with the reduction in 60S subunits in the mutant. We did not observe misincorporated ribosomal proteins or biogenesis factors among nascent 60S particles in *fkbp39Δ* cells. This suggests that the primary role of 60S subcompartmentalization is to promote efficient subunit assembly, including RNA domain III incorporation. Fkbp39 interacts directly only with nascent 60S, but the disruption in the nucleolar organization and the chromatin accumulation of nascent 60S in *fkbp39Δ* cells is likely to indirectly affect processing of the 18S rRNA, causing some delay in the initial stages of 40S biogenesis, but those subunits will eventually complete their biogenesis. Cross-talk between the 60S and 40S processing has been observed before: the depletion of a factor involved in early 60S maturation (Rat1 exonuclease) caused delays in 40S processing, as evidenced by the accumulation of 35S pre-rRNA.^40^ Although the mechanism for such cross-talk remains to be established, these data indicate that sub-compartmentalization of nascent 60S and 40S is required for their proper biogenesis.

In closing, we show that chromatin localization mediates specific partitioning of nucleophosmin with nascent 60S and subsequent sub-compartmentalization of their biogenesis into the GC (Figure 7). The scenario *in vivo* is undoubtedly more complex, with additional factors involved, but nonetheless, our work highlights the importance of protein-chromatin interactions in the assembly of ribosomal subunits and nucleolar organization.

### Limitations of the study

Our data reveal that Fkbp39 is localized on chromatin and separates nascent 60S from chromatin and from nascent 40S. The data suggest that this occurs through LLPS. Nonetheless, the importance of phase separation in these processes remains to be determined. We show that Fkbp39 is enriched over 25S rDNA, and our data suggest the involvement of transcription in Fkbp39 localization; however, it remains unclear how Fkbp39 is specifically enriched over this region.

## STAR★METHODS

### RESOURCE AVAILABILITY

#### Lead contact

Further information and requests for resources and reagents should be directed to and will be fulfilled by the lead contact, Mario Halic (Mario.Halic@stjude.org).

#### Materials availability

Plasmids and strains generated in this study are available upon request.

#### Data and code availability

- EM densities have been deposited in the Electron Microscopy Data Bank and PDB under accession codes EMD-24395 and PDB:8ETI (Figure 2, state 1), EMD-24396 and PDB:8ETJ (Figure 2, state 2), EMD-24397 and PDB:8ETG (Figure 2, state 3), EMD-24398 and PDB:8ETC (Figure 2, state 4), EMD-24409 and PDB:8ETH (Figure 5, state 1B), EMD-24410 and PDB:8EUP (Figure 5, state 1A), EMD-24411 and PDB:8ESQ (Figure 5, state 2), EMD-24412 and PDB:8EUG (Figure 5, state 3), EMD-24420 and PDB:8EUY (Figure S6I, state 1A), EMD-24421 and PDB:8EV3 (Figure S6I, state 1B), EMD-24422 and PDB:8ESR (Figure S6I, state 2), EMD-24423 and PDB:8EUI (Figure S6I, state 3).
- The sequencing data that support the findings of this study have been deposited in the National Center for Biotechnology Information Gene Expression Omnibus (GEO) and are accessible through the GEO Series Accession Number GSE156203. All data needed to evaluate the conclusions in the paper are present in the paper and/or the supplementary information. Accession numbers are also listed in the key resources table.
- This paper does not report original code.
- Any additional information required to reanalyze the data reported in this paper is available from the lead contact upon request.

### EXPERIMENTAL MODEL AND SUBJECT DETAILS

All yeast and bacterial strains used in this study are listed in the key resources table and are available upon request.

### METHOD DETAILS

#### Strain construction and plasmid generation

All *S. pombe* strains used in this study are listed in Table S7. The strains were generated by PCR-based gene targeting as described previously.^41^ Plasmids and oligos used in this study are listed in Tables S8 and S9 respectively. For bacterial overexpression, fkbp39 and fkbp41 were each cloned into a pETDuet vector with a 6xHis-SUMO N-terminal tag followed by an HRV-3C recognition site. Mutant fkbp39 and fkbp41 constructs were generated via inverse PCR of this plasmid.

#### Chromatin immunoprecipitation qPCR and sequencing

ChIP was performed as described in Marasovic et al.^41^ Briefly mid-log phase yeast cultures were cross linked with 1% formaldehyde for 15 minutes at room temperature and quenched with 125 mM glycine. The RNase-treated samples were crosslinked for 5 minutes according to Abruzzi et al.^42^ The pellet was resuspended in lysis buffer (250 mM KCl, 1X Triton x-100, 0.1% SDS, 0.1% Na-Desoxycholate, 50 mM Hepes pH 7.5, 2 mM EDTA, 2 mM EGTA, 5 mM MgCl2, 0.1% Nonidet P40, 20% glycerol) with 1 mM PMSF and complete EDTA-free Protease Inhibitor Cocktail (Roche). Cells were disrupted by mechanical lysis with BioSpec Fast-Prep-24 bead beater and the DNA sheared via sonication. Cell debris was removed by centrifugation and the normalized crude lysate was incubated over night at 4°C with the immobilized antibody (HA-probe Santa Cruz Biotechnology immobilized on Dynabeads Protein A, Thermo Scientific; anti-FLAG M2 Magnetic Beads Sigma). The resin was extensively washed with lysis buffer and the DNA-protein complex eluted with 150 μl of elution buffer (50 mM Tris HCl pH 8, 10 mM EDTA, 1% SDS) at 65°C for 15 minutes. Both inputs (normalized crude lysate aliquots) and elutions were de-crosslinked (95°C for 15 minutes) and treated with RNase A (Invitrogen) and Proteinase K (Thermo Scientific) at 37°C over night. DNA was recovered by phenole-chloroform-isoamylalcohol (25:24:1, Carl Roth) extraction followed by ethanol precipitation. DNA was analyzed by qPCR with rDNA specific primers, the fold enrichment was normalized to input levels (primers are listed in Table S9). Alternatively, ChIP seq libraries were prepared using the NEBNext Ultra II DNA Library Prep Kit for Illumina (NEB) following the manufacturer instructions. When an RNase treatment step was added to the ChIP protocol, chromatin from the same experiment was treated with 5 U of RNase A (20 mg/ml, Invitrogen) or an equivalent volume of RNase storage buffer (50 Mm Tris-HCl pH8.0, 10 mM EDTA). After incubating at room temperature for 30 minutes, immunoprecipitations were performed as described above.

#### Endogenous protein purification

10 L of yeast cultures OD 1 were pelleted and resuspended in lysis buffer (100 mM NaCl, 7.5 mM MgCl2, Na Phosphate pH 8, 0.1% Nonidet P40, 5% glycerol) supplemented with 1 mM DTT, 1 mM PMSF and Complete EDTA-free Protease Inhibitor Cocktail (Roche) in a 1:5 buffer:pellet ratio. The suspension was dripped in liquid nitrogen to create small frozen drops suitable for cryogenic grinding by Freezer Mill (SPEX Sample Prep), alternatively cells were disrupted by mechanical lysis with FastPrep-24 (MP Biomedicals) bead beater. The powder pellet was thawed and lysis buffer without glycerol was added to reach a final buffer:pellet ratio of 1:1. The lysate was incubated either with Pierce universal nuclease for Cell lysis (Thermo Fisher) or DNase I (Thermo Scientific, in this case the buffer was supplemented with 130 μM CaCl2) for 30 minutes at 4°C on rotation. Cell debris was removed by centrifugation and the normalized crude lysate was incubated with 100 μl anti-FLAG M2 affinity gel (Sigma) for 3 hours at 4°C on rotation. The beads were extensively washed with lysis buffer without glycerol and with elution buffer (100 mM NaCl, 7.5 mM MgCl2, 100 mM sucrose, Na Phosphate pH 8). Elution was performed three times adding 150 μl of elution buffer supplemented with 3XFLAG peptide (Sigma, final concentration 150 μg/ml) to the beads, incubated for 20 minutes shaking at 4°C. The protein content was analyzed on denaturing acrylamide gel. The samples were eventually used for cryo-EM grids preparation and mass spectrometry analysis (Proteomics Core Facility, EMBL Heidelberg and Proteomics Facility, St. Jude Children’s Research Hospital, Memphis).

#### In vivo co-immunoprecipitation

50 ml of pelleted yeast cells were resuspended in lysis buffer (100 mM NaCl, 5 mM MgCl2, 50 mM Tris pH 7.5, 0.1% Nonidet P40, 5% glycerol) supplemented with 1 mM DTT, 1 mM PMSF and Complete EDTA-free Protease Inhibitor Cocktail (Roche), Pierce universal nuclease for Cell lysis (Thermo Fisher) and disrupted by mechanical lysis with BioSpec FastPrep-24 bead beater. Cell debris was removed by centrifugation and the normalized crude lysate was incubated with 10 μl anti-FLAG M2 affinity gel (Sigma) for 3 hours at 4°C on rotation. The beads were extensively washed with lysis buffer. Elution was performed two times adding 15 μl of lysis buffer supplemented with 3XFLAG peptide (Sigma, final concentration 150 μg/ml) to the beads, incubated for 20 minutes shaking at room temperature. The protein content was analyzed by western blot (Monoclonal ANTI-FLAG M2-Peroxidase (HRP) antibody Sigma, HA-probe Santa Cruz Biotechnology, Anti-Mouse IgG-Peroxidase Sigma), Goat Anti-Mouse IgG (H+L)-HRP conjugate antibody, BIO-RAD).

#### Polysome profiles

100 ml of yeast culture OD 1 was pelleted, frozen and then resuspended in lysis buffer (100 mM KOAc, 7.5 mM Mg(OAc)2, 125 mM sucrose, 20 mM Hepes pH 7.5) supplemented with 1 mM DTT, 1 mM PMSF and Complete EDTA-free Protease Inhibitor Cocktail (Roche). Cells were lysed by glass bead disruption using BioSpec FastPrep-24 bead beater and cells debris was removed by centrifugation. Between 5–10 260 nm absorbance unit of the normalized samples were loaded onto a linear 10–50% sucrose gradient (100 mM KOAc, 7.5 mM Mg(OAc)2, 20 mM Hepes pH 7.5, 1 mM DTT) and centrifuged for 3 hours onto SW40 Beckman Coulter rotor at 40K rpm. For cycloexhimide treatment, cells were incubated with 0.1 mg/ml cycloexhimide for 5 minutes prior to pelleting. All the further steps were performed identically, but the solutions were supplemented with 0.1 mg/ml cycloexhimide. Gradients were collected on a Gradient Station (Biocomp Instruments) with an Econo UV Monitor (BIO-RAD) and a FC203B Fraction Collector (Gilson). For the quantification of the 260 nm signal, the polysome profiles from different strains were aligned and the peaks corresponding to different species were identified. For each strain the signal from the void fraction (non ribosome) and the ribosome fractions (everything but the void) was normalized to the signal of the whole gradient.

#### Nascent RNA labeling and RNA library preparation

For nascent RNA labeling, a yeast over night culture was diluted in YEA media and let grow up to OD 600 nm 0.6. 4 Thiouracil (Sigma) was supplemented to 5 mM final concentration, and aliquots for RNA extraction were taken after 2 and 10 minutes (and before 4 Thiouracil addition).^43^ Cells were briefly centrifuged and immediately frozen in liquid nitrogen. RNA was isolated following the hot phenol method as described in Brönner et al.^44^ Briefly the pellet was resuspended in 500 μl lysis buffer (300 mM NaOAc pH 5.2, 10 mM EDTA, 1% SDS) and 500 ml phenol-chloroform-isoamylalcohol (25:24:1, Carl Roth) and incubated at 65°C for 10 minutes, mixing. The aqueous fraction was separated from the organic fraction by centrifugation at 20000 x g for 10 minutes and then ethanol precipitated. The nucleic acids were treated with DNAse I (Thermo Scientific) for 2 hours at 37°C and the RNA was recovered with a second phenol-chloroform-isoamylalcohol extraction. 30 μg of RNA were treated with EZ-Link HPDP-Biotin (Thermo Scientific) to biotin-label the 4 Thiouracil -SH group. The reaction was carried on in 500 ml of biotinylation buffer (100 mM Tris pH 7.5, 10 mM EDTA) and 300 μg/ml EZ-Link HPDP-Biotin for 2 hours at room temperature shaking in a thermomixer. RNA was recovered by phenolchloroform-isoamylalcohol extraction followed by ethanol precipitation. The nascent RNA (biotin labeled) can be isolated thanks to the interaction biotin-streptavidin. Prior to binding, streptavidin beads (Roche) were blocked with a non specific single stranded DNA sequence (hph cassette sequence) in binding buffer (100 mM NaCl, 10 mM Tris pH 7.5, 1 mM EDTA) for 20 minutes shaking in a thermomixer at room temperature. A spike in DNA sequence (mouse Ahctf1 sequence, coding for ELYS protein) was added to the beads, which were then distributed to the RNA samples and incubated for 15 minutes shaking in a thermomixer at room temperature. The beads were washed 6 times with washing buffer (1 M NaCl, 100 mM Tris pH 7.5, 10 mM EDTA, 0.1% tween), the first 3 performed at 65°C. Biotinylated RNA was eluted twice with 100 mM DTT for 5 minutes shaking in a thermomixer at room temperature and ethanol precipitated. The recovered RNA was used for the preparation of RNA library using the NEBNext Ultra II Directional RNA Library Prep Kit for Illumina (NEB) following the manufacturer instructions.

For steady state RNA library, RNA was extracted following the hot phenol method, treated with DNAse I (Thermo Scientific) and used for the preparation of RNA library using the NEBNext Ultra II Directional RNA Library Prep Kit for Illumina (NEB) following the manufacturer instructions.

#### Dot blot

After treatment with EZ-Link HPDP-Biotin (Thermo Scientific), 500 ng of heat denatured (for 10 minutes at 65°C) total RNA were spotted on an Amersham Hybond-N+ positively charged nylon membrane (Cytiva). The RNA was ultraviolet-cross-linked to the membrane with Spectrolinker XL-1500 (Spectroline, ‘optimal crosslink’) and blocked for 10 minutes in blocking solution (PBS pH 7.5, 10% SDS, 1 mM EDTA). The membrane was then incubated in 1:1000 dilution of streptavidin-HRP (Pierce) in blocking buffer for 15 minutes. After washings with decreased concentration of SDS, the signal was recorded (Amersham ECL Western Blotting Detection Kit, Cytiva).

#### Growth curve

Cells were grown in YES rich media. Overnight cultures were diluted in the morning to OD 0.2 and let recover for about 1 hour. The OD value was then measured every two hours. In the evening the cultures were diluted to avoid saturation (dilution factor 1:100), the OD was then measured again in the morning. For the mixed population experiment, after recovering in the morning, wild type and *fkbp39Δ* (G418 resistant) cells were mixed in approximately 1:1 cells ratio and plated on YES plates (between 100–1000 cells). The mixed culture was grown for a total of three days, each day it was diluted to avoid saturation and about 100–1000 cells were plated on YES plates. After colonies appeared on YES plates, cells were replica-plated on G418-YES selective plates. Plates were imaged and the number of colonies on selective and non-selective media counted.

#### Analysis of sequencing data

Single end sequencing of libraries was performed on an Illumina GAIIX sequencer (Illumina) at the LAFUGA core facility of the Gene Center, Munich. The Galaxy platform was used todemultiplex the obtained reads with Je-Demultiplex-Illu.^45,46^ Demultiplexed illumina reads were mapped to the *S. pombe* genome, allowing 2 nucleotides mismatch using NovoAlign (Novocraft, https://www.novocraft.com/products/novoalign/). The Fkbp39, Fkbp41 and Ytm1 ChIP seq datasets were normalized to regions, which were not changed in the different strains (background). Steady state RNA and nascent RNA datasets were normalized to total reads. The background contamination for nascent RNA libraries was assumed uniformly distributed within each sample, and so eliminated after internal normalization. Custom perl scripts were used for reads assignment, extractions and quantification.^41,47^

We used the genome sequence and annotation available from the *S. pombe* Genome Project.^48^ The data are displayed using the Integrative Genomics Viewer (IGV).^49^ Sequenced strains are listed in Table S10.

#### Over expressed protein purification and labeling

Fkbp39 constructs were overexpressed in *E. coli* Rosetta with 0.2 mM IPTG at 18°C overnight. Pelleted cells were resuspended in lysis buffer (500 mM NaCl, 20 mM imidazole, 50 mM Hepes pH 7.5) supplemented with 3 mM β-mercaptoethanol, 1 mM PMSF and lysozyme and lysed by sonication. The clear supernatant was incubated with Ni Sepharose 6 Fast Flow resin (Cytiva) for 30 minutes at 4°C. The resin was washed with lysis buffer and with 4 bed volumes of washing buffer (500 mM NaCl, 40 mM imidazole, 50 mM Hepes pH 7.5, 3 mM β-mercaptoethanol). The bound protein was eluted with 5 bed volumes of elution buffer (350 mM NaCl, 300 mM imidazole, 35 mM Hepes pH 7.5, 3 mM β-mercaptoethanol). The protein tag was proteolytically removed during overnight dialysis at 4°C against 300 mM NaCl, 30 mM Hepes pH 7.5, 3 mM β-mercaptoethanol. In order to remove the tag, the protein was incubated again with Ni Sepharose 6 Fast Flow resin (Cytiva) for 25 minutes at 4°C (imidazole was adjusted to 20 mM). The protein was further purified via ion exchange (SP column, Cytiva) using a linear gradient of salt starting from 250 mM NaCl in 30 mM Hepes pH 7.5 1 mM DTT (the protein eluted around 600mM NaCl).

Fkbp41 was overexpressed and purified using a similar work-flow. After overnight overexpression in *E. coli* Rosetta (DE3) by induction with IPTG, pelleted bacteria were resuspended in lysis buffer 1 M NaCl, 10% glycerol, 20 mM imidazole-Cl pH 8, 50 mM HEPES-Na pH 8 supplemented with 3 mM *β*ME, 2 mM PMSF, Pierce Universal Nuclease for Cell Lysis (Thermo Scientific), and lysozyme then sonicated. After centrifugation, the clarified supernatant was incubated with Ni Sepharose 6 Fast Flow resin for 20–30 minutes at 4°C then washed with lysis buffer followed by 4 bed volumes of wash buffer 1 M NaCl, 10% glycerol, 40 mM imidazole-Cl pH 8, 50 mM HEPES-Na pH 8 supplemented with 3 mM *β*ME. Resin-bound protein was eluted with 4 bed volumes elution buffer 1 M NaCl, 10% glycerol, 200 mM imidazole-Cl pH 8 supplemented with 3 mM βME. After adding protease, the eluted protein was dialyzed overnight at 4°C against 30 mM HEPES-Na pH 8, 3 mM *β*ME, 0.1 mM EDTA, and an NaCl concentration such that the final salt concentration after dialysis was 150 mM NaCl. The cleaved tag was removed by adjusting the imidazole concentration to 20 mM and incubating again with Ni Sepharose 6 Fast Flow resin at 4°C. The protein was then purified by ion exchange chromatography (IEX) on a HiTrap Q Fast Flow column (Cytiva) using a linear salt gradient starting from 150 mM NaCl in 30 mM HEPES-Na pH 8 and 1 mM DTT.

Fkbp39 and Fkbp41 were at times further purified by size exclusion chromatography (SEC) on a Superdex 200 Increase 10/300 GL column (Cytiva) over an isocratic elution spanning one bed volume of the column. The column was equilibrated in buffer 30 mM HEPES-Na pH 8, 150 mM NaCl, 1 mM DTT (or 0.5 mM TCEP) or buffer 10 mM Tris-Cl pH 7.5, 300 NaCl, 1 mM DTT (or 0.5 mM TCEP).

For turbidity assays the protein was either eluted during SEC or dialyzed against buffer 300 mM NaCl, 10 mM Tris pH 7.5 or 8, 1 mM DTT (or 0.5 mM TCEP).

For fluorescent microscopy experiments, an additional cysteine was cloned at Fkbp39 C terminus since Fkbp39 native cysteine is buried within the nucleophosmin oligomerization domain and therefore not accessible for labeling. This mutant was purified as described above, with the only difference that 0.5 mM TCEP replaced DTT during ion exchange. For fluorescent cysteine labeling the protein was concentrated to 20 μM and incubated with CF 405S dye (Biotium, Fremont, CA) for two hours at room temperature following the manufacturer instructions. Alternatively for phase diagram generation, the protein was labeled using Alexa Fluor 546 (Invitrogen). The labeling reaction was quenched with excess DTT. The protein was dialyzed 4 times against 300 mM NaCl, 10 mM Tris pH 7.5, 1 mM DTT to remove unreacted dye. A mixture of labeled and non labeled Fkbp39 was concentrated for fluorescence microscopy experiments.

#### Histone octamer preparation

Wild type *Xenopus laevis* histones were over-expressed in BL21(DE3) pLysS cell strain and purified from inclusion bodies. Shortly, pelleted cells were resuspended in wash buffer (50 mM Tris-HCl, pH 7.5, 100 mM NaCl, 1 mM EDTA, 1 mM DTT) and sonicated. After centrifugation (20 min, 17000 rpm at 4°) the pellet was re-suspended in wash buffer supplemented with 1% (v/v) Triton X-100, sonicated once more and spun down. The inclusion bodies were re-suspended in wash buffer supplemented with Triton x-100 and recovered by centrifugation. This step was repeated 2 more times, using wash buffer without Triton. The purified inclusion bodies, one for each histone protein, were incubated for 1 hour at room temperature in 50 mM Tris pH 7.5, 2 M NaCl, 6 M guanidine hydrochloride, 1 mM DTT and insoluble components were removed by centrifugation. H2A-H2B, and H3-H4 were combined in equimolar ratios and dialyzed two times in 1L of refolding buffer (25 mM HEPES/NaOH, pH 7.5, 2 M NaCl, 1 mM DTT) at +4 °C, at least one dialysis step was done over night. Any precipitate was removed by centrifugation. The soluble histone pairs were further purified via cation-exchange chromatography in batch (SP Sepharose Fast Flow resin), as described in Bilokapic and Halic.^50^ Soluble histone pairs were concentrated and purified by size exclusion chromatography equilibrated in 25 mM HEPES/NaOH, pH 7.5, 2 M NaCl, 1 mM DTT. Clean protein fractions were pooled, concetrated and used for histone octamer assembly. Shortly, 2.5 fold excess of H2A/H2B histone dimer was mixed with H3/H4 histone tetramer and incubated over-night at +4°C. The excess of the H2A/H2B dimer was purified from the histone octamer by size exclusion chromatography (Superdex 200 Increase 10/300 GL) pre-equilibrated in 25 mM HEPES/NaOH pH 7.5, 2 M NaCl, 1 mM DTT.

#### Nucleosomal DNA preparation

The DNA for nucleosome assembly (either containing 40 bp overhangs on each side of the centrally positioned 601 DNA sequence, 227bp, or the 147 bp 601 sequence) was PCR amplified from a plasmid,^51^ (primers are listed in Table S9). The DNA was recovered by ethanol precipitation, resuspended in 15 mM HEPES/NaOH pH 7.5, 2 M NaCl, 1 mM DTT and used for nucleosome assembly.^52^

#### Nucleosome assembly

Nucleosome assembly was done by the ‘double bag’ dialysis method, as described in Bilokapic and Halic^50^ and Bilokapic et al.^53^ The purified histone octamer^54^ and the DNA were mixed in a 1:1 ration into a dialysis button, placed inside a dialysis bag filled with 50 ml of 15 mM HEPES/NaOH pH 7.5, 2 M NaCl, 1 mM DTT. The dialysis bag was initially dialyzed against 1L of 15 mM HEPES/NaOH pH 7.5, 1M NaCl, 1 mM DTT over-night at + 4°C, then against 1L of 15 mM HEPES/NaOH pH 7.5, 50 mM NaCl, 1 mM DTT for 5 hours. Finally the dialysis button, released from the dialysis bag, was dialyzed for 1 hour against 15 mM HEPES/NaOH pH 7.5, 50 mM NaCl, 1 mM DTT. The quality of the reconstituted nucleosomes was assessed by 6% native PAGE.

For fluorescence microscopy experiments, nucleosomes were assembled as described above using fluorescent labeled DNA (PCR amplified using Alexa 647 labeled primers, IDT). A mixture of labeled and non labeled nucleosomes was used for fluorescence microscopy experiments.

#### Nascent 60S subunits labeling

Nascent 60S subunits purified from *S. pombe* through Fkbp39-FLAG endogenous protein purification, were incubated over night with a 100 fold excess of single stranded Atto 488 labeled DNA oligos (IDT) complementary to the 25S rRNA at 4°C (Table S9). In order to remove non bound oligos, the complex was dialyzed against 100 mM NaCl, 7.5 mM MgCl2, 50 mM Na phosphate pH 8.

#### In vitro binding assay

Fkbp39 purified from *E. coli* over-expression culture was transferred to incubation buffer (100 mM NaCl, 5 mM MgCl2, 50 mM Na phosphate pH 8, 20% glycerol, 1 mM DTT for cysteine mutant) via PD-10 desalting column (Cytiva). Increasing amount of Fkbp39 were incubated with DNA, RNA, nucleosomes, nascent 60S subunits or nascent 40S subunits at 20 °C for 30 minutes in incubation buffer. The complex formation was analyzed on 6% TBE acrylamide gel (Fkbp39:DNA; Fkbp39:RNA; Fkbp39:nucleosome complex) or on 0.8% TBE agarose gel (Fkbp39:nascent 60S subunits complex, Fkbp39:nascent 40S subunits complex).

For competition experiments, Fkbp39 was pre-incubated with either nucleosomes, DNA or nascent 60S subunits at 20 °C for 20 minutes in incubation buffer, then the other component was added and the reaction incubated for further 20 minutes The reaction was analyzed both on 6% TBE acrylamide gel and 0.8% TBE agarose gel to follow Fkbp39:nucleosome or Fkbp39:DNA and Fkbp39:nascent 60S subunits complexes formation. All the gels were stained with SYBR Gold (ThermoFisher Scientific).

For binding assays involving nascent 60S subunit and either Fkbp39 or Fkbp41, increasing concentrations of Fkbp39 or Fkbp41 were first equilibrated in 5 mL containing incubation buffer with 50 mM HEPES-Na pH 8 instead of sodium phosphate pH 8 for 20 min at 20°C. Reactions were then mixed with 5 mL, equilibrated at 20°C prior to mixing, containing nascent 60S subunits and reaction buffer. Mixed reactions were further incubated for 30 min at 20°C before complex formation analysis by 1x TBE, 0.8% agarose gel electrophoresis. Electrophoresis was run at 50 volts for 2 hours at 4°C in 1x TBE running buffer. Running buffer was exchanged for fresh running buffer at 30-minute intervals during the run. For cooperative nascent 60S subunit binding assays, increasing Fkbp39 concentrations were first incubated with or without constant or increasing concentrations of Fkbp41.

Nascent 60S subunits and nascent 40S subunits mole determination carries a certain degree of uncertainty. Nascent subunits are indeed a mixture of different species not completely characterized and their mole calculation (estimated from RNA absorbance) carries about 1.5 fold uncertainty. This is applicable to all the experiments with nascent subunits and is particularly relevant for competition assays (for which mole ratios have to be considered approximate).

#### In vitro Co-immunoprecipitation

Incubated 12 μg each 6xHis-SUMO-Fkbp39 and Fkbp41 either individually or together for 45 min at 4 C° in 60 μL containing reaction buffer 30 mM HEPES-Na pH 7.5–8, 150 mM NaCl, 1 mM DTT, and 1.5 mM βME supplemented with 20 mM imidazole-Cl pH 8. These incubations were mixed with 40 μL of a ~50% Ni S6 FF resin slurry equilibrated in reaction buffer supplemented with imidazole-Cl and incubated further for 25 min at 4 C°. Resins were collected by passing samples over Micro Bio-Spin Columns (BIO-RAD) and washed with 4 bed volumes of reaction buffer supplemented with imidazole-Cl. Eluted resin-bound protein with 4 bed volumes of elution buffer 20 mM HEPES-Na 7.5–8, 100 mM NaCl, 666 nM DTT, 1 mM βME supplemented with ~350 mM imidazole-Cl pH 8. Assessed complex formation by SDS-PAGE analysis of flow-through, wash, and elution fractions alongside 1.2 μg each protein (10% of that used in reactions) on 4–15% TGX Mini-PROTEAN gels (BIO-RAD). Samples were electrophoresed at 300 volts for 15 minutes running in 1x SDS buffer. Gels were stained using SimplyBlue SafeStain (Invitrogen).

#### Turbidity assay

Dilutions of Fkbp39 (362C) or Fkbp41 (363C) were prepared in 125 mM NaCl, 10 mM Tris-Cl (pH 7.5), and 0.5 mM TCEP in a final volume of 15 μL. Solutions were incubated approximately 10 min before 12.5 μL of each solution was transferred to a 384-well clear flat bottom microplate (Corning). Turbidity was determined by measuring UV-Vis absorbance at 340 nm using a POLARstar Omega microplate reader (BMG LABTECH).

#### Fluorescence microscopy

Fkbp39:nucleosomes condensates were formed by mixing 5 μM Fkbp39 labeled with CF 405S (Biotium, San Fransisco, CA) with 1.3 pmole of nucleosomes labeled with Alexa Fluor 647 (Carlsbad, CA) in 7.5 mM Tris, 12.5 mM sodium phosphate, 137.5 mM NaCl, 2 mM MgCl2, pH 8.0. Fkbp39:nascent 60S subunits condensates were formed by mixing 5 μM Fkbp39 labeled with CF 405S dye with 1.3 pmol of nascent 60S subunits labeled with Atto 488 dye (Sigma-Aldrich, St. Louis, MO) in 7.5 mM Tris, 12.5 mM sodium phosphate, 137.5 mM NaCl, 2 mM MgCl2, pH 8.0. Condensates were transferred to a 16-well CultureWell chambered slide (Grace BioLabs, Bend, OR) coated with PlusOne Repel Silane ES (Cytiva, Pittsburgh,PA) and Pluronic F-127 (Sigma-Aldrich, St. Louis, MO) and allowed to equilibrate for an hour at room temperature. To test for displacement of nucleosomes by nascent 60S subunits in Fkbp39:nucleosome condensates, 5 μM of CF 405S labeled Fkbp39 and 1.3 pmol of Alexa 647 labeled nucleosomes were mixed and allowed to equilibrate at room temperature for one hour. To these condensates, 1.3 pmol of labeled nascent 60S subunits were added. Condensates were imaged after one hour. Partition coefficients were determined for all components. To test for the displacement of nascent 60S subunits by nucleosomes, Fkbp39:n60S condensates were prepared by mixing 5 mM labeled Fkbp39 and 1.3 pmol of labeled nascent 60S subunits and allowed to equilibrate at room temperature for one hour. To these 1.3 pmol of labeled nucleosomes were added. Condensates were incubated for an hour and imaged. Partition coefficients were determined for all components. As a controls for dilution, the same volume of buffer was added to pre-formed Fkbp39:nucleosomes or Fkbp39:nascent 60S subunits condensates and imaged using the same parameters.

Condensates were imaged using an LSM 780 NLO point scanning confocal microscope (Carl Zeiss Microscopy GmbH, Jena, Germany) with 63x Plan Apochromat (N.A. 1.4) objective. Imaging parameters were adjusted to offset any fluorescence signals from buffer alone.

Partition coefficients were calculated using the equation: (I_dense_/I_light_)/C.F., where I_dense_ is the mean intensity inside condensates, I_light_ is the mean intensity outside the condensates and C.F. is the correction factor for the differences in quantum yields of the each dye in highly viscous environments.^31^

Condensates across the entire image (67.5 μm x 67.5 μm) were identified using default intensity threshold settings on FIJI image processing software.^55^ To determine the fluorescence outside the condensates, intensity thresholds were manually set to remove signals from out of focus condensates. Partition coefficients for each component were measured for more than 300 condensates and averaged over four imaging fields per condition.

Since nascent 60S subunits are bigger than nucleosomes, they may contain more binding sites for Fkbp39. Because of this, we normalized nascent 60S subunits and nucleosomes partition coefficients with respect to their masses.

Nascent 60S subunits mole was calculated measuring the RNA absorbance, and assuming that all the intermediates contain full length 25S rRNA (1 mole of 25S rRNA is equal to 1 mole of n60S). Nascent 60S subunits mass was calculated based on cryo-EM data, as an average of the different class masses related to their abundance. Since in cryo-EM flexible regions are not visible, strong hits from mass spectrometry experiments non visible in the structures were also taken into account for mass calculation. We estimated nascent 60S subunits to be about 9.7 times bigger in mass than nucleosomes. Moles and mass for nascent 60S subunits are approximate, they should be considered with at least 1.5 fold uncertainty.

To generate the phase diagrams Fkbp39 labeled with Alexa 546 was mixed with either DNA or rRNA (labeled with Atto 488) in buffer containing NaCl > 300mM to prevent pre-formed homotypic Fkbp39 condensates. Salt-free buffer was added to the solutions to a final buffer composition of 7.5 mM Tris, 12.5 mM sodium phosphate, 137.5 mM NaCl, 2 mM MgCl_2_ pH 8.0. DNA was fluorescently labeled with SYBR Gold Nucleic Acid dye (Invitrogen). Solutions were imaged using an LSM 780 NLO point scanning confocal microscope (Carl Zeiss Microscopy GmbH, Jena, Germany) with 63x Plan Apochromat (N.A. 1.4) objective.

#### CryoEM grid preparation and data collection

3 μl of nascent 60S subunits, purified from *S. pombe* through Fkbp39-FLAG or FLAG-Ytm1 endogenous protein purification, were applied to freshly glow-discharged Quantifoil R3.5/1 2nm carbon grid (Electron Microscopy Sciences). Humidity in the chamber was kept at 95% and temperature at + 10 °C. After 40s binding time, the grids were blotted for 1s and plunge-frozen in the liquid ethane using FEI Vitrobot automatic plunge freezer.

Electron micrographs were recorded on FEI Titan Krios (ThermoFisher Scientific) at 300 kV with a Gatan K3 Summit electron detector at the Cryo-EM facility at St. Jude Children’s Research Hospital. We recorded ~9 000 images for Fkbp39 IP, 8 000 images for Ytm1 IP from wild type cells and ~15 000 images for Ytm1 IP from *fkbp39* deletion cells. Image pixel size was 1.06 Å per pixel on the object scale. Data were collected in a defocus range of 4 000 Å – 15 000 Å with a total exposure of 90 e/Å^2^. 50 frames were collected and aligned with the MotionCor2 software using a dose filter.^56,57^ The contrast transfer function parameters were determined using CTFFIND4.^58^

Several thousand particles were manually picked and used for training and automatic particle picking in crYOLO.^59^ Particles were windowed and 2D class averages were generated with the Relion software package.^60^ Inconsistent class averages were removed from further data analysis. The initial reference was filtered to 40 Å in Relion. Particles were split into 2 datasets and refined independently and the resolution was determined using the 0.143 cut-off (Relion auto refine option). All maps were filtered to resolution using Relion with a B-factor determined by Relion. Initial 3D refinement were done with all particles, followed by classification of various conformational states. All maps were refined to final resolutions between 2.8 Å and 3.8 Å and were filtered to local resolution. Structures of nascent 60S subunits (PDB: 3JCT, 6EM1, 6ELZ, 6C0F, 6CB1)^33,35,61^ were docked into our maps and used to segregate and assign densities.

The model of *S. cerevisiae* pre-60S particle state E (PDB:6ELZ)^33^ was fitted as a rigid body to state 3 cryo-EM map of Ytm1 pull-down from *fkbp39Δ* cells due to the best resolution of the map (Figures 5A and S6E). This served as an initial model for fitting ribosomal RNA, ribosomal proteins and biogenesis factors. 3D structures of *S. pombe* ribosomal proteins and biogenesis factors were predicted by Alphafold^62^ and fitted into cryoEM map replacing *S. cerevisiae* proteins. Protein assignment was confirmed by side chain density and secondary structure pattern. Protein and rRNA modifications were done manually in Coot^63^ and the overall model refined in Phenix using real_space_refinement.^64,65^ Models for other maps were created based on the refined model of state 3 cryoEM map of Ytm1 pull-down from *fkbp39Δ* cells. First, a general model was created by trimming segments and removing chains not represented by the corresponding density maps, additional biogenesis factors were added into each map as needed, protein loops and rRNA segments were manually built using Coot. The models were refined in Phenix using real_space_refinement.

Visualization of all cryo-EM maps and models was done with Chimera.^66^

### QUANTIFICATION AND STATISTICAL ANALYSIS

All of the statistical details of experiments can be found in the figure legends, including the statistical tests used, exact value of n, and dispersion and precision measures (SEM).

## Supplementary Material

MMC2

MMC1

## Figures and Tables

**Table 1.** Cryo-EM data

	Fkbp39 statel	Fkbp39 state2	Fkbp39 state3	Fkbp39 state4	Ytm1(wt) state 1A	Ytm1(wt) state 1B
PDB code	8ETI	8ETJ	8ETG	8ETC	8ETH	8EUP

EMD code	EMD-24395	EMD-24396	EMD-24397	EMD-24398	EMD-24410	EMD-24409

Data collection and processing						

Magnification						
Voltage (kV)	300	300	300	300	300	300
Electron exposure (e^−^/Å^2^)	60	60	60	60	60	60
Defocus range (μm)	−0.7 to −2.5	−0.7 to −2.5	−0.7 to −2.5	−0.7 to −2.5	−0.7 to −2.5	−0.7 to −2.5
Pixel size (Å)	1.06	1.06	1.06	1.06	1.06	1.06
Symmetry imposed	C1	C1	C1	C1	C1	C1
Initial particle images (no.)	~167,000	~167,000	~167,000	~167,000	~376,000	~376,000
Final particle images (no.)	~9,000	~9,000	~9,000	~9,000	~14,000	~71,000
Map resolution (Å) FSC threshold	3.7	3.2	3.4	3.1	3.8	3.1
Map resolution range (Å)	3.5–20.0	3.0–20.0	3.2–20.0	2.8–20.0	3.5–20.0	2.8–20.0

Refinement						

Initial model used	6ELZ	6ELZ	6ELZ	6ELZ	6ELZ	6ELZ

Model composition						

Nonhydrogen atoms	91,105	77,914	97,665	95,104	78,722	77,577
Protein residues	7,648	4,979	7,472	6,145	6,156	6,167
Nucleotides	1,841	1,907	2,120	2,298	1,623	1,508
Ligands	1	1	1	1	1	1

<B> factors (Å^2^)						

Protein	39.93	61.9	34.89	59.00	37.48	9.09
Nucleotides	55.90	85.5	37.18	72.33	46.96	28.28
Ligand	20.86	84.34	23.15	62.45	47.51	17.25

RMS deviations						

Bond lengths (Å)	0.006	0.006	0.003	0.003	0.005	0.006
Bond angles (°)	0.864	0.757	0.638	0.604	0.555	0.617

Validation						

MolProbity score	2.17	2.20	1.98	1.97	1.94	1.89
Clashscore	11.13	19.41	14.70	13.63	12.20	10.12
Rotamer outliers (%)	1.85	0.92	0.57	0.72	0.4	0.41

Ramachandran plot						

Favored (%)	94.0	93.64	95.58	95.37	95.82	94.65
Allowed (%)	5.78	6.15	4.31	4.37	5.29	5.11
Disallowed (%)	0.23	0.21	0.11	0.27	0.15	0.22

	Ytm1(wt) state 2	Ytm1(wt) state 3	Ytm1(fkbp39Δ) state 1A	Ytm1(fkbp39Δ) state 2	Ytm1(fkbp39Δ) state 1B	Ytm1(fkbp39Δ) state 3

PDB code	8ESQ	8EUG	8EUY	8ESR	8EV3	8EUI

EMD code	EMD-24411	EMD-24412	EMD-24420	EMD-24422	EMD-24421	EMD-24423

Data collection and processing						

Magnification						

Voltage (kV)	300	300	300	300	300	300
Electron exposure (e^−^/Å^2^)	60	60	60	60	60	60
Defocus range (μm)	−0.7 to −2.5	−0.7 to −2.5	−0.7 to −2.5	−0.7 to −2.5	−0.7 to −2.5	−0.7 to −2.5
Pixel size (Å)	1.06	1.06	1.06	1.06	1.06	1.06
Symmetryimposed	C1	C1	C1	C1	C1	C1
Initial particle images (no.)	~376,000	~376,000	~683,000	~683,000	~683,000	~683,000
Final particle images (no.)	~9,000	~9,000	~220,000	~109,000	~250,000	~59,000
Map resolution (Å) FSC threshold	2.8	2.8	3.0	3.2	3.0	3.1
Map resolution range (Å)	2.5–20.0	2.6–20.0	2.7–20.0	3.0–20.0	2.7–20.0	2.8–20.0

Refinement						

Initial model used	6ELZ	6ELZ	6ELZ	6ELZ	6ELZ	6ELZ

Model composition						

Nonhydrogen atoms	128,450	123,362	80,193	117,293	87,614	123,668
Protein residues	10,254	7,144	6,141	9,443	6,603	7,239
Nucleotides	2,532	3,133	1,629	2,371	1,671	3,139
Ligands	1	1	1	1	1	1

<B> factors (Å^2^)						

Protein	24.99	13.2	18.48	34.74	17.34	12.92
Nucleotides	23.30	17.71	28.52	53.75	32.32	21.73
Ligand	30.33	5.06	63.68	16.16	21.37	30.53

RMS deviations						

Bond lengths (Å)	0.004	0.009	0.006	0.005	0.004	0.005
Bond angles (°)	0.734	1.020	0.774	0.765	0.615	0.799

Validation						

MolProbity score	2.52	1.79	2.23	2.25	1.80	2.28
Clashscore	38.57	9.18	17.32	33.77	11.37	34.53
Rotamer outliers (%)	1.39	0.02	1.68	0.41	0.21	0.24

Ramachandran plot						

Favored (%)	95.11	95.68	95.35	96.41	96.48	96.19
Allowed (%)	4.62	4.12	4.21	3.46	3.32	3.68
Disallowed (%)	0.27	0.20	0.44	0.13	0.19	0.13

**Table:** KEY RESOURCES TABLE

REAGENT or RESOURCE	SOURCE	IDENTIFIER
Antibodies		

Mouse Monoclonal Anti-HA-Probe Antibody (F-7) X	Santa Cruz Biotechnology	Cat#sc-7392 X
Mouse Monoclonal Anti-FLAG^®^ M2 Antibody Magnetic Beads	Millipore	Cat#M8823
Mouse Monoclonal ANTI-FLAG^®^ M2 Antibody Affinity Gel	Millipore	Cat#A2220
Mouse Monoclonal ANTI-FLAG^®^ M2-Peroxidase (HRP) Antibody	Sigma-Aldrich	Cat#8592
*Goat Anti-Mouse IgG (H + L)-HRP Conjugate*	BIO-RAD	Cat#1706516

Bacterial and virus strains		

Rosetta^™^ Competent Cells – Novagen^®^	Sigma-Aldrich	Cat#70953
Rosetta^™^ (DE3) Competent Cells – Novagen^®^	Sigma-Aldrich	Cat#70954
BL21(DE3) pLysS Competent Cells	Agilent	Cat#200132
Biological samples		

Chemicals, peptides, and recombinant proteins		

Dynabeads^™^ Protein A for Immunoprecipitation	Invitrogen^™^	Cat#10002D
Roti^®^ Phenol/Chloroform/Isoamyl alcohol	Carl Roth	Cat#A156
PureLink^™^ RNase A (20mg/mL)	Invitrogen^™^	Cat#12091021
Proteinase K, recombinant, PCR Grade	Thermo Scientific^™^	Cat#EO0491
Pierce^™^ Universal Nuclease for Cell Lysis	Thermo Scientific^™^	Cat#88702
DNase I, RNase-free (1 U/μL)	Thermo Scientific^™^	Cat#EN0521
3X FLAG^®^ Peptide	Sigma-Aldrich	Cat#F4799
4-Thiouracil	Sigma-Aldrich	Cat#440736
EZ-Link^™^ HPDP-Biotin	Thermo Scientific^™^	Cat#21341
Streptavidin Magnetic Particles	Roche	Cat#STREPMAG-RO
Alexa Fluor 546 C_5_ Maleimide	Invitrogen	Cat#A10258
CF^®^405S Dye Maleimide	Biotium	Cat#92030
PlusOne Repel Silane ES	Cytiva	Cat#17133201 https://www.cytivalifesciences.com/en/us/shop/protein-analysis/electrophoresis-and-isoelectric-focusing/reagents-for-electrophoresis-and-ief/plusone-repel-silane-es-p-01199
Pluronic F-127	Sigma-Aldrich	Cat#P2443 https://www.sigmaaldrich.com/US/en/product/sigma/p2443

Critical commercial assays		

NEBNext^®^ Ultra^™^ II DNA Library Prep Kit for Illumina	New England BioLabs^®^ Inc.	Cat#E7645
NEBNext^®^ Ultra^™^ II Directional RNA Library Prep Kit for Illumina^®^	New England Biolabs^®^ Inc.	Cat#E7760
Amersham ECL Western Blotting Detection Kit	Cytiva	Cat# RPN2108

Deposited data		

Figure 2, State 1	This Paper	EMD-24395
Figure 2, State 2	This Paper	EMD-24396
Figure 2, State 3	This Paper	EMD-24397
Figure 2, State 4	This Paper	EMD-24398
Figure 5, State 1A	This Paper	EMD-24409
Figure 5, State 1B	This Paper	EMD-24410
Figure 5, State 2	This Paper	EMD-24411
Figure 5, State 3	This Paper	EMD-24412
Figure S7, State 1A	This Paper	*EMD-24420*
Figure S7, State 1B	This Paper	EMD-24421
Figure S6, State 2	This Paper	EMD-24422
Figure S6, State 3	This Paper	EMD-24423
See Table S10 for Sequencing Data	This Paper	GSE156203

Experimental models: Organisms/strains		

See Table S7 for *S. pombe* strains used in this study		N/A

Oligonucleotides		

See Table S9 for oligos used in this study		N/A

Recombinant DNA		

See Table S8 for plasmids used in this study		N/A

Software and algorithms		

Galaxy	Goecks et al., 2010	https://usegalaxy.org/
*Je demultiplex-illu*	Girardot et al., 2016	https://gbcs.embl.de/portal/tiki-index.php?page=Je
NovoAlign	Novocraft	https://www.novocraft.com/products/novoalign/
Integrative Genomics Viewer	Thorvaldsdó ttir et al., 2013	https://software.broadinstitute.org/software/igv/
FIJI	Schindelin et al., 2012	https://imagej.net/software/fiji/
MotionCor2	Zheng et al., 2017	https://emcore.ucsf.edu/ucsf-software
CTFFIND4	Rohou and Grigorieff, 2015	https://grigoriefflab.umassmed.edu/ctf_estimation_ctffind_ctftilt
crYOLO	Wagner et al., 2019	https://sphire.mpg.de/wiki/doku.php?id=pipeline:window:cryolo
RELION	Scheres, 2012; Zivanov et al., 2018	https://www3.mrc-lmb.cam.ac.uk/relion/index.php/Main_Page
AlphaFold	Jumper et al., 2021	https://github.com/deepmind/alphafold
Coot	Emsley et al., 2010	https://www2.mrc-lmb.cam.ac.uk/personal/pemsley/coot/
PHENIX	Afonine et al., 2018; Liebschner et al., 2019	https://phenix-online.org/
Chimera	Pettersen et al., 2004	https://www.rbvi.ucsf.edu/chimera/

Other		

QUANTIFOIL^®^ R 3.5/1 2 nm carbon grid	Electron Microscopy Sciences	https://www.emsdiasum.com/microscopy/products/grids/quantifoil.aspx
Amersham Hybond-N+	Cytiva	https://www.cytivalifesciences.com/en/us/shop/protein-analysis/blotting-and-detection/nylon-membranes/amersham-hybond-n-p-05398
CulturWell ChamberSLIP 16, Non-Removeable Chambered Coverglass	Grace Bio-Labs	SKU:112359 http://gracebio.com/product/culturewell-chamberslip-16-with-non-removable-chambered-coverglass-112359/
Freezer Mill	Spex^®^ SamplePrep	https://www.spexsampleprep.com/
*Fast Prep 24 Bead Beating Grinder*	MP Biomedicals	SKU:116004500 https://www.mpbio.com/us/fastprep-24-classic-instrument-1-each
Gradient Station^™^	BioComp Instruments	https://www.biocompinstruments.com/
Econo UV Monitor	BIO-RAD	https://www.bio-rad.com/
FC 203B Fraction Collector	Gilson	SKU:171011 https://www.gilson.com/fc203b-110-220v.html
Spectrolinker^™^ XL-1500 UV Crosslinker	Spectroline^™^	Mfr Cat#XL-1500 Fisher Scientific Cat#11–992-90 https://www.fishersci.com/shop/products/microprocessor-controlled-uv-crosslinkers/1199290
Genome Analyzer_*IIX*_	Illumina^®^	Cat#SY-301–1301
Pombase	Wood et. al., 2002; Harris et al., 2021	https://www.pombase.org/
ÄKTA pure chromatography system	Cytiva	https://www.cytivalifesciences.com/en/us/shop/chromatography/chromatography-systems/akta-pure-p-05844
POLARStar Omega Plate Reader	BMG LABTECH	https://www.bmglabtech.com/en/
LSM 780 NLO	Carl Zeiss/Zeiss	https://www.zeiss.com/microscopy/en/home.html
FEI Titan Krios	ThermoFisher Scientific	https://www.thermofisher.com/de/en/home/electron-microscopy.html
K3 Summit Electron Detector	GATAN	https://www.gatan.com/
FEI Vitrobot Mark IV	ThermoFisher Scientific	https://www.thermofisher.com/us/en/home/electron-microscopy/products/sample-preparation-equipment-em/vitrobot-system.html

## Highlights

- Fkbp39 associates with rDNA encoding the 60S ribosomal subunit
- Fkbp39 binds nascent 60S, promoting its partition from chromatin and from nascent 40S
- Fkbp39 directs the outward translocation of 60S intermediates within the nucleolus
