# Supplementary material for: Chromatin localization of nucleophosmin organizes ribosome biogenesis
Source: Mol Cell. Author manuscript; Available in PMC 2023 Feb 23. (PMC9949351; doi:10.1016/j.molcel.2022.10.033)
Supplement: MMC1 [file NIHMS1849199-supplement-MMC1.pdf]

Molecular Cell, Volume *82*

Supplemental information

Chromatin localization of nucleophosmin organizes ribosome biogenesis

Ilaria Ugolini, Silvija Bilokapic, Mylene Ferrolino, Josiah Teague, Yinxia Yan, Xuelin Zhou, Ashish Deshmukh, Michael White, Richard W. Kriwacki, and Mario Halic

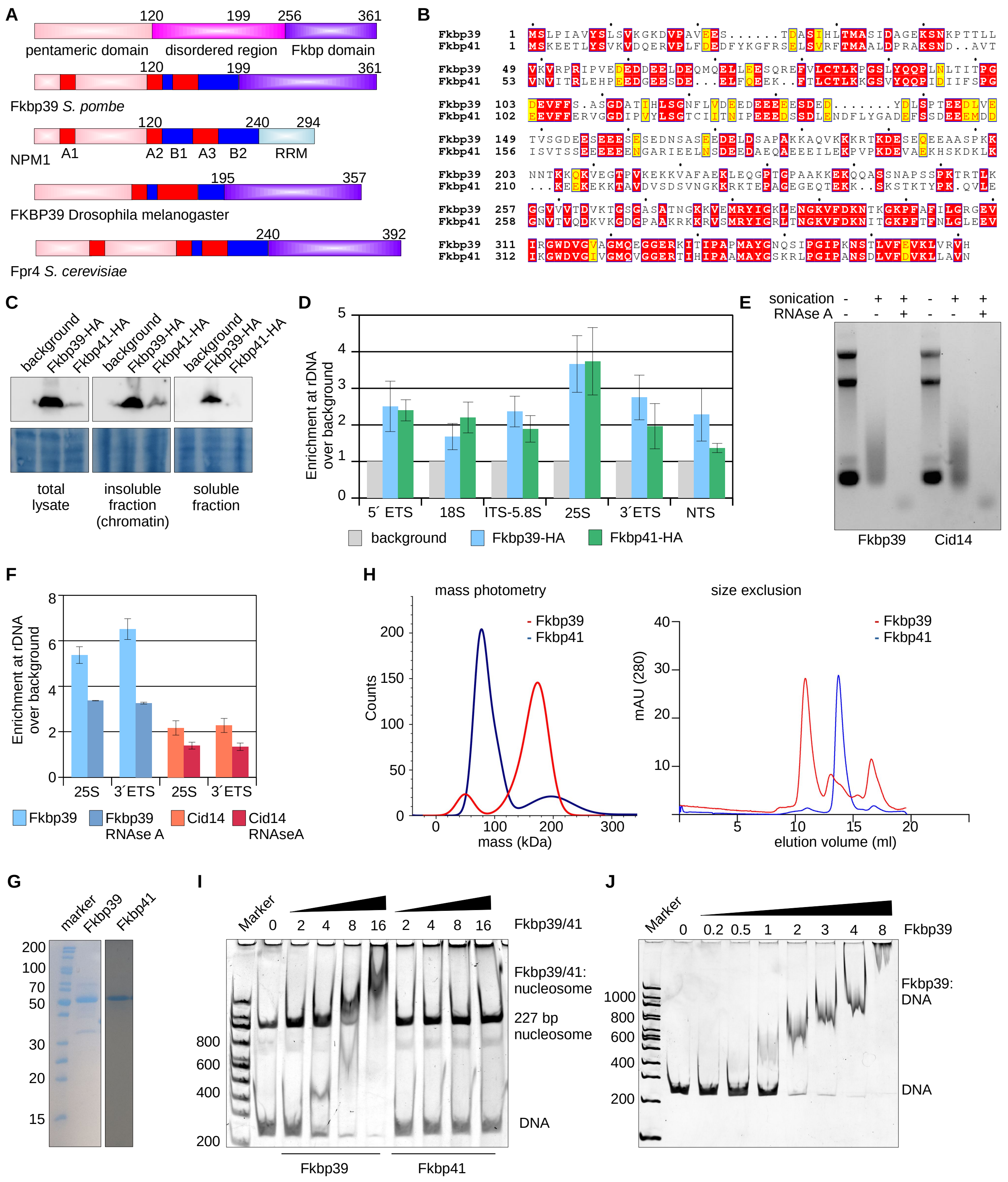

Suppl. Figure 1

Figure S1: Fkbp39 binds to open chromatin, Related to Figure 1.

A, Schematic domain organization of NPM1 and members of the NPL-FKBP family of proteins. The N terminal pink domain represents the NPL oligomerization domain (responsible for pentamerization), followed by the intrinsically disordered region, depicted in lilac, composed by acidic and basic tracts (in red and blue respectively). Plant and yeast nucleophosmin proteins have a C-terminal prolyl isomerase Fkbp domain, instead of the nucleic acid–binding domain RRM found in vertebrates. NPM1 C-terminal nucleic acid binding domain, RRM, is shown in light blue, whereas the C-terminal FKBP prolyl isomerase domains are depicted in violet.

B, Alignment of *S. pombe* Fkbp39 and Fkbp41. Conserved residues are highlighted in red, residues with similar properties in yellow. The alignment was generated with the ESPript server (http://espript.ibcp.fr).

C, Western blot showing Fkbp39 and Fkbp41 expression levels in wild type cells. Wild type cells expressing either Fkbp39-HA or Fkbp41-HA were lysed and the total, insoluble (chromatin) and soluble fractions were analyzed by anti-HA western blot. The amido black stained membrane is showed as loading control. This is a representative image from four independent experiments.

D, Quantification of nucleophosmin ChIP-seq reads over the rDNA locus. Quantification is the average of four independent ChIP-seq experiments for Fkbp39 and three for Fkbp41 and error bars represent the standard error. Reads were normalized to background regions and plotted relative to background.

E, Native gel stained by Sybr Green II showing RNA in Fkbp39 and Cid14 ChIP experiments before and after RNase A treatment.

F, Quantification of Fkbp39 and Cid14 enrichment over rDNA locus by ChIP-qPCR with and without Rnase A treatment. Quantification is the average of three independent ChIP experiments and error bars represent the standard error.

G, Representative SDS gels of the purified proteins used for *in vitro* binding assays.

H, Mass photometry and gel filtration showing migration of Fkbp39 and Fkbp41.

I, *In vitro* binding assay showing that Fkbp39 binds 227 bp DNA or nucleosomes with 40 bp symmetrical linker DNA. Fkbp41 does not bind either DNA nor nucleosome with 40 bp symmetrical linker DNA. The relative molar ratio of Fkbp39 and Fkbp41 in respect to nucleosomes is indicated, considering their functional unit a pentamer. The formation of the complexes was visualized on 6% TBE acrylamide gel stained with SYBR gold. This is a representative experiment of three independent assays.

J, *In vitro* binding assay showing that Fkbp39 binds 227 bp DNA. The relative molar ratio of Fkbp39 in respect to DNA is indicated, with Fkbp39 functional unit being a pentamer. The formation of the complex was visualized on 6% TBE acrylamide gel stained with SYBR gold. This is a representative experiment of three independent assays.

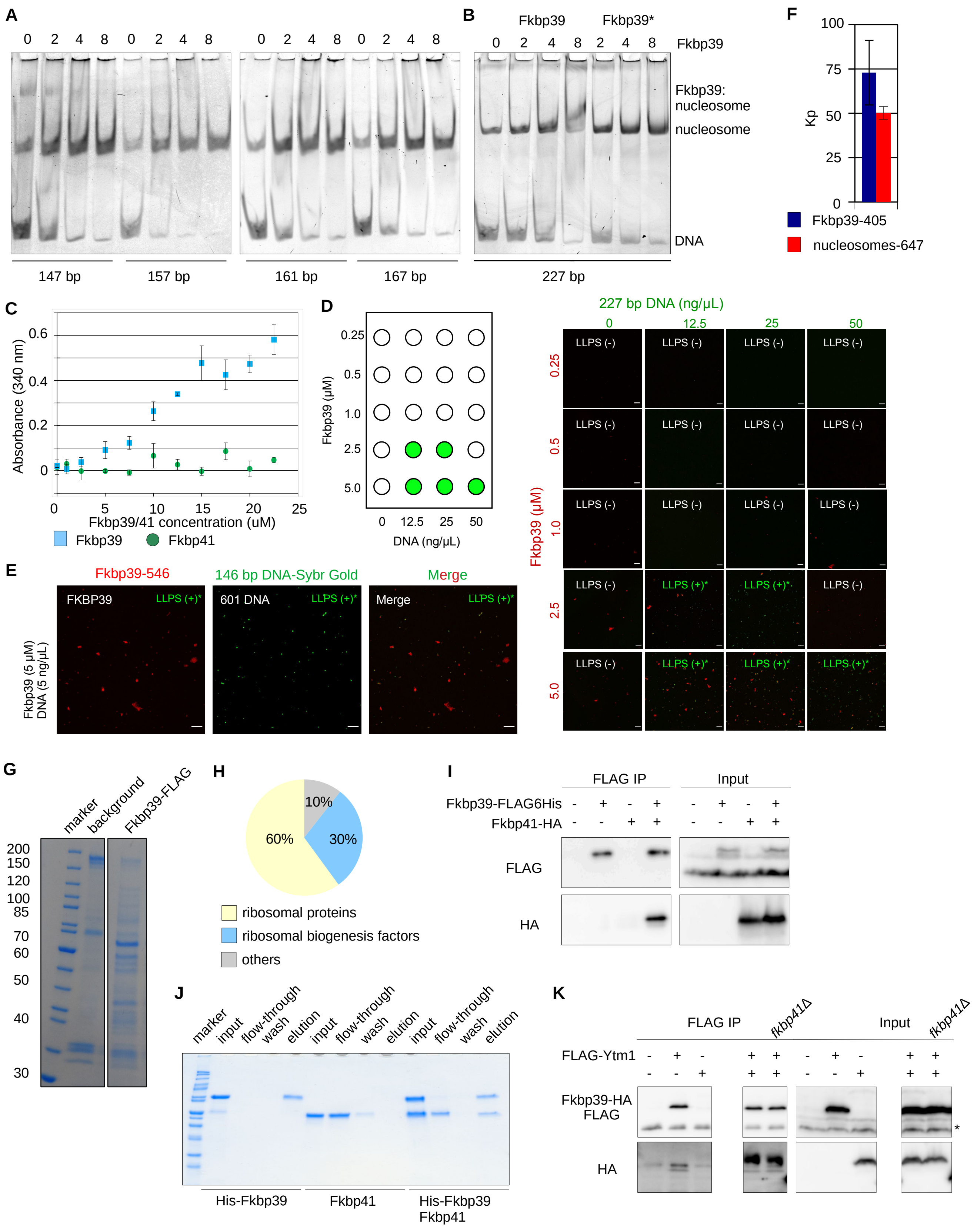

Suppl. Figure 2

Figure S2: Fkbp39 binds chromatin and nascent ribosomes, Related to Figure 1.

A, *In vitro* binding assay showing that Fkbp39 does not bind the nucleosome core particle (147bp DNA) or nucleosomes with a symmetrical linker DNA up to 10 bp (167 bp). The relative molar ratio of Fkbp39 in respect to nucleosomes is indicated, with Fkbp39 functional unit being a pentamer. The formation of the complex was visualized on 6% TBE acrylamide gel stained with SYBR gold. This is a representative experiment of three independent assays.

B, *In vitro* binding assay showing that Fkbp39 F301C/W314C/Y337K (Fkbp39*) mutant does not bind nucleosome. The relative molar ratio of Fkbp39 in respect to nucleosomes is indicated, with Fkbp39 functional unit being a pentamer. The formation of the complex was visualized on 6% TBE acrylamide gel stained with SYBR gold. This is a representative experiment of three independent assays.

C, Turbidity assay showing that Fkbp39 undergoes homotypic phase separation, whereas Fkbp41 does not. Serial dilutions of Fkbp39 and Fkbp41 (X axis) were incubated and the light scattering at 340 nm (Y axis) was measured. Error bars represent standard deviation from measurements of three independent experiments.

D, Right, confocal fluorescence microscopy images of Fkbp39:DNA condensates mixed at various concentrations. Fkbp39 labeled with alexa 546 was incubated with 227 bp DNA. DNA was stained non specifically by Sybr Green I before imaging. Scale bars represents 10 µm. Left, visualization of phase diagram; concentrations with heterotypic condensates are shown in green.

E, Confocal fluorescence microscopy images of Fkbp39:DNA condensates. 5 µM Fkbp39 labeled with alexa 546 was incubated with 5 ng/µl 227 bp DNA. DNA was stained non specifically by Sybr Green I before imaging. Scale bars represents 10 µm.

F, Partition coefficients (Kp) related to Figure 1D. The partition coefficients for each component were determined from more than 300 condensates, error bars represent standard deviation.

G, Representative SDS gel showing the Fkbp39-FLAG purification analyzed by mass spectrometry and used for structural analysis by cryo-EM (Figure 2A and S3).

H, Pie chart depicting the functional classification of Fkbp39 co-immunoprecipitating proteins from the mass spectrometry results shown in Table S1.

I, Co-immunoprecipitation experiment between Fkbp39-FLAG6His and Fkbp41-HA. Immunoprecipitates and inputs were analyzed by anti-FLAG and anti-HA western blot. This is a representative experiment of two independent replicates.

J, *In vitro* binding assay between His-Fkbp39 and Fkbp41. Each step of binding assay and inputs were analyzed by coomasie staining. This is a representative experiment of two independent replicates.

K, Co-immunoprecipitation experiment between FLAG-Ytm1 and Fkbp39-HA in wild type cells. Immunoprecipitates and inputs were analyzed by anti-FLAG and anti-HA western blot. The asterisk represents antibody non specific signal. This is a representative experiment of three independent replicates.

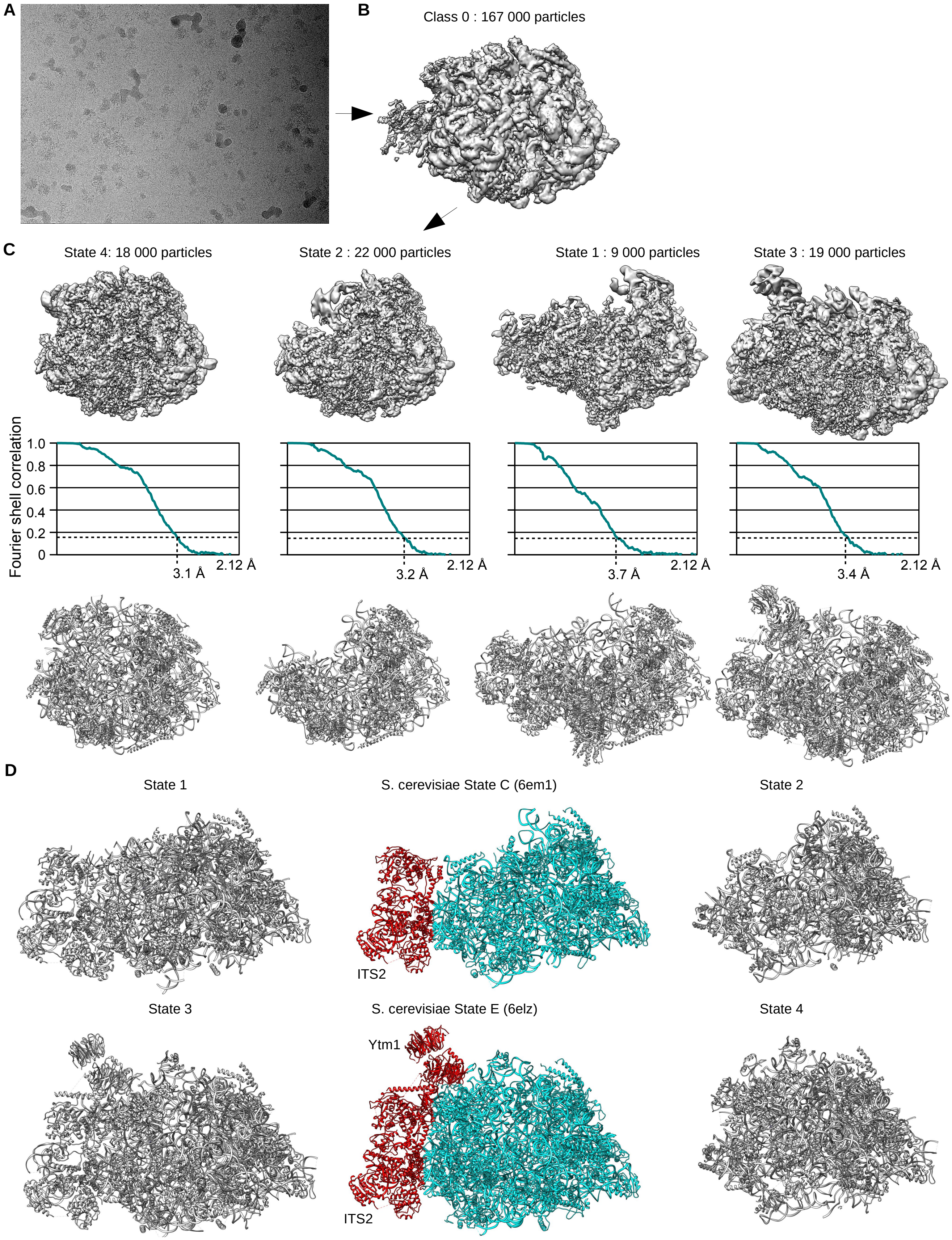

Suppl. Figure 3

Figure S3: Cryo-EM analysis of Fkbp39 interacting particles, Related to Figure 2.

A, Representative cryo-EM micrograph collected with Titan Krios electron microscope at 300 keV.

B, Initial map generated from the entire dataset comprising 167 000 particles. The dataset was further extensively classified.

C, Cryo-EM maps of nascent 60S interacting with Fkbp39. Maps, filtered to the corresponding local resolution, are shown on the left. The fourier shell correlation (FSC) curve showing the resolution of the map is shown in the center. Model for each map is shown on the right.

D, Comparison of S. pombe states 1-4 (gray) with S. cerevisiae states C (6em1) and E (6elz) (cyan). ITS2 foot is colored red.

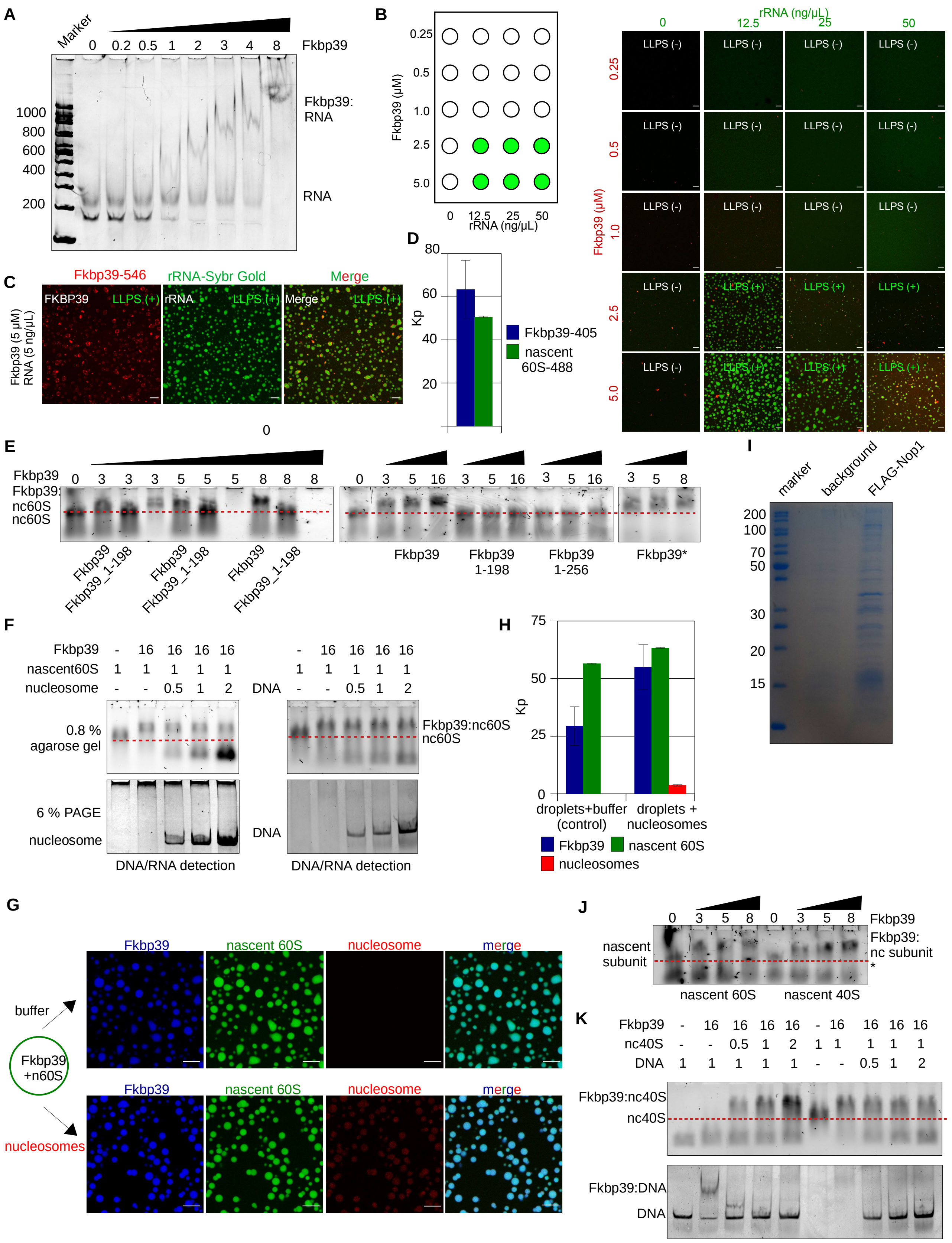

Suppl. Figure 4

Figure S4: Fkbp39 binds and forms condensates with nascent 60S, Related to Figure 3.

A, *In vitro* binding assay showing that Fkbp39 binds 130bp RNA. The relative molar ratio of Fkbp39 in respect to RNA is indicated, with Fkbp39 functional unit being a pentamer. The formation of the complex was visualized on 6% TBE acrylamide gel stained with SYBR gold. This is a representative experiment of three independent assays.

B, Right, confocal fluorescence microscopy images of Fkbp39:RNA condensates mixed at various concentrations. Fkbp39 labeled with alexa 546 was incubated with S. pombe rRNA. RNA was stained non specifically by Sybr Green II before imaging. Scale bars represents 10 µm. Left, visualization of phase diagram; concentrations with heterotypic condensates are shown in green.

C, Confocal fluorescence microscopy images of Fkbp39:RNA condensates. 5 µM Fkbp39 labeled with alexa 546 was incubated with 5 ng/µl rRNA. RNA was stained non specifically by Sybr Green II before imaging. Scale bars represents 10 µm.

D, Partition coefficients (Kp) related to Figure 2C. The partition coefficients for each component were determined from more than 300 condensates, error bars represent standard deviation.

E, *In vitro* binding assay showing that Fkbp39 prolyl isomerase domain binds nascent 60S subunits but the prolyl isomerase activity is dispensable for binding. The relative molar ratio of Fkbp39 in respect to nascent 60S subunits is indicated, with Fkbp39 functional unit being a pentamer, although the prolyl isomerase domain (Fkbp39_199-361) which lacks the NPL oligomerization domain does not form pentamers. Fkbp39*, activity mutant, has the following mutations F301CW314CY337K. The formation of the complex was visualized on 0.8% TBE agarose gel stained with SYBR gold. The red dash line indicates the position of nascent60S. This is a representative experiment of three independent assays.

F, *In vitro* binding assay showing that Fkbp39 does not dissociate from nascent 60S subunits. Increasing amount of nucleosomes (left) or DNA (right) were added to preformed Fkbp39:nascent60S complex. The relative molar ratio of each component is indicated, with Fkbp39 functional unit being a pentamer. Nucleosome and DNA complexes are visualized on 6% native PAGE, whereas Fkbp39:nascent60S on 0.8% TBE agarose gel. This is a representative experiment of two independent assays.

G, Confocal fluorescence microscopy images showing that nucleosomes can not replace nascent 60S in Fkbp39:nascent60S condensates. Nucleosomes or buffer were added to pre-equilibriated Fkbp39:nascent60S condensates. Fkbp39 is labeled with CF 405, nucleosomes with Alexa 647 and nascent 60S with Atto 488. Scale bars represent 10 µm. These are representative images from one of two independent experiments.

H, Partition coefficients (Kp) related to Figure S4G. The partition coefficients for each component were determined from more than 300 condensates, error bars represent standard deviation. The Kp values for nascent 60S and nucleosomes were normalized to their mass (described in details in Methods).

I, Representative SDS gel from the FLAG-Nop1 purification analyzed by mass spectrometry.

J, In vitro binding assay showing Fkbp39 binding to nascent 40S subunits. The relative molar ratio of Fkbp39 in respect to nascent subunits is indicated, with Fkbp39 functional unit being a pentamer. The formation of the complex was visualized on 0.8% TBE agarose gel stained with SYBR gold. The red dash line represents the migration line of nascent subunits. This is a representative experiment of three independent assays. The asterisk marks a non-specific band.

K, *In vitro* binding assay showing that Fkbp39 dissociates from DNA to bind nascent 40S subunits, but does not dissociate from nascent 40S subunits to bind DNA. Increasing amount of nascent 40S subunits were added to preformed Fkbp39:DNA complexes (left). Increasing amount of DNA (right) were added to preformed Fkbp39:nascent40S complex. The relative molar ratio of each component is indicated, with Fkbp39 functional unit being a pentamer. Fkbp39:DNA complexes are visualized on 6% native PAGE, whereas Fkbp39:nascent40S on 0.8% TBE agarose gel. This is a representative experiment of three independent assays.

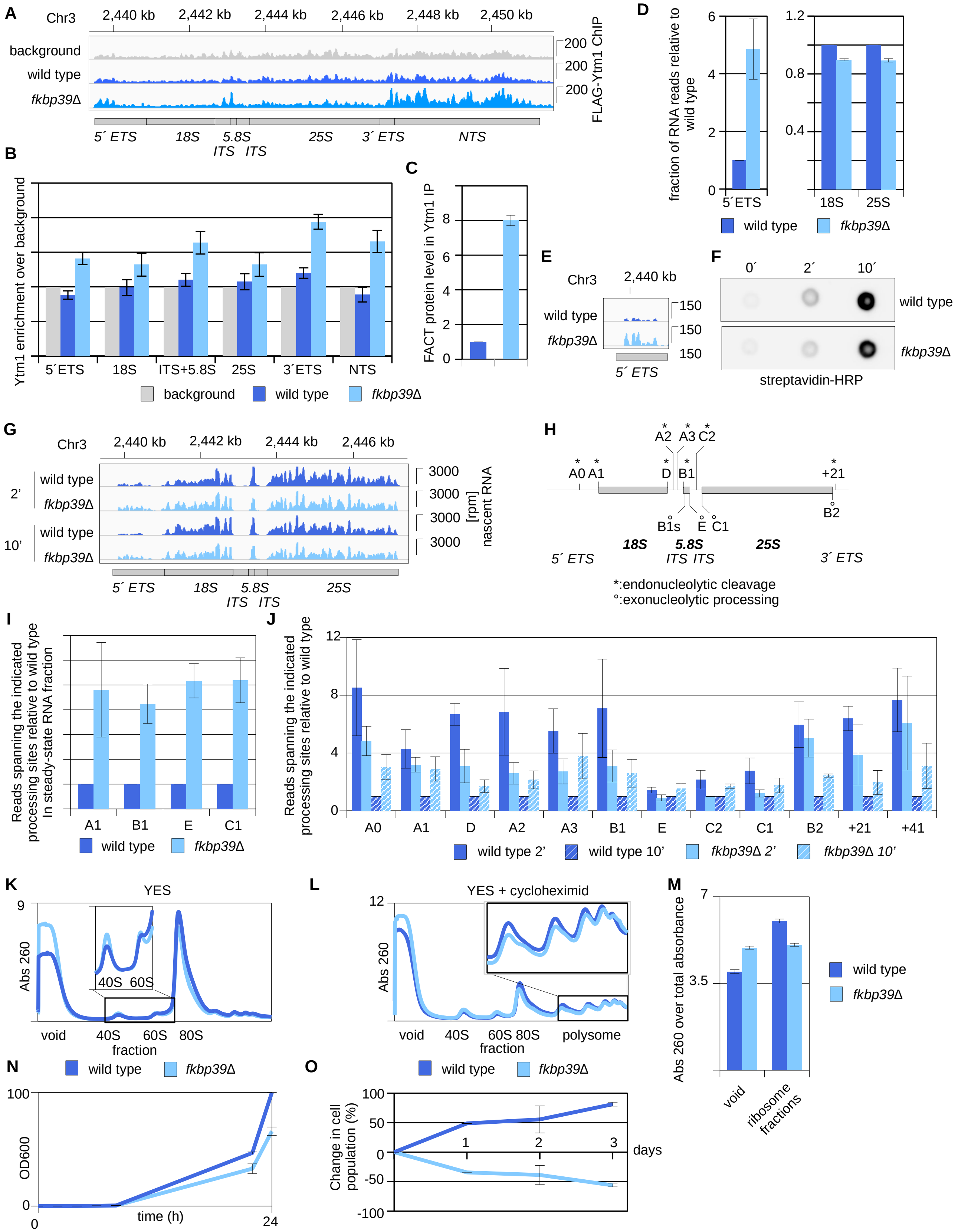

Suppl. Figure 5

Figure S5: Defects in rRNA processing and ribosome assembly in *fkbp39Δ* cells, Related to Figure 4.

A, Ytm1 ChIP-seq in wild type and *fkbp39Δ* cells. Ytm1 is detected on the rDNA locus only in *fkbp39Δ* cells, mainly over the 3'ETS and the non transcribed spacer (NTS). Scale bars on the right denote read numbers per million reads (rpm) normalized over background regions. The coordinates of the genomic location are indicated above the chart, the rDNA locus organization is depicted below in gray boxes.

B, Quantification of the reads mapping over the rDNA locus from Ytm1 ChIP-seq experiments (Figure S5A). Reads were normalized to background regions and plotted relative to background. Quantification is based on six independent ChIP-seq experiments, error bars represent standard error.

C, Quantification of mass spectrometry data of Ytm1 immunoprecipitation from wild type and *fkbp39Δ* cells showing that Ytm1-containing nascent 60S interact with histone chaperone FACT in *fkbp39Δ* cells. Peptide counts of the indicated proteins were normalized to total spectral counts and plotted relative to peptide counts in wild type cells. Quantification is from 2 independent mass spectrometry experiments, error bars represent standard deviation. A complete list of the mass spectrometry results is available in supplementary materials.

D, Quantification of the reads mapping over the rDNA locus from RNA-seq experiments in the indicated strains. Quantification is based on two independent, error bars represent standard error.

E, Visualization of total RNA-seq (+ strand) data from the indicated strains. Scale bars on the right denote read numbers per million reads (rpm). The coordinates of the genomic location are indicated above the chart, the rDNA locus organization is depicted below in gray boxes.

F, Dot blot showing incorporation of 4-Thiouracil into RNA after 2 and 10 minutes of 4 thiouracil labeling. Purified RNA was treated with EZ-Link HPDP-Biotin to biotin-label the 4 Thiouracil -SH group and spotted on the membrane. The biotin-label RNA is detected with HRP conjugated- streptavidin.

G, Nascent RNA (+ strand) from the indicated strains. Scale bars on the right denote read numbers per million reads (rpm). The coordinates of the genomic location are indicated above the chart, the rDNA locus organization is depicted below in gray boxes.

H, Schematic representation of rRNA processing sites in yeast.

I, Quantification of sense reads spanning the indicated rRNA processing sites in total RNA. Sense reads spanning the indicated processing sites were quantified and normalized to the amount of rRNA sense transcripts, wild type value was set to 1. Quantification from two independent total RNA sequencing, error bars represent standard error.

J, Quantification of sense reads spanning the indicated rRNA processing sites in nascent RNA. Sense reads spanning the indicated processing sites were quantified and normalized to the amount of rRNA sense transcripts, wild type 10 minutes time point value was set to 1. Quantification from three independent nascent RNA sequencing, error bars represent standard error.

K, Representative polysome profile experiment performed without cycloheximide. 40S and 60S subunits fractions are shown as zoomed in panel. The X axis represents the sucrose gradient, from 10% on the left to 50% on the right. The 260 nm absorbance signal is plotted on the Y axis.
L, Polysome profile experiment in presence of cycloheximid. The polysome fraction is shown as zoomed in panel. The X axis represents the sucrose gradient, from 10% on the left to 50% on the right. The 260 nm absorbance signal is plotted on the Y axis.
M, Quantification of the 260 nm signal from the polysome profile experiments performed without cycloheximid. Quantification from 5 independent polysome profiles, error bars represent standard error (see Methods for a detailed description).
N, Growth curve for wild type and *fkbp39Δ* yeast cells. Time in hour is plotted on the x axis, cells amount (represented by the optical density, O.D., at 600 nm) is on the y axis. Wild type OD was arbitrary set to 100 after a growth of 24 hours. Average from two independent growth curve experiments, error bars represent standard deviation.
O, Growth curve showing distribution of wild type and *fkbp39Δ* yeast cells in a mixed population. Fraction of *fkbp39Δ* yeast cells was determined based on their resistance to G418.

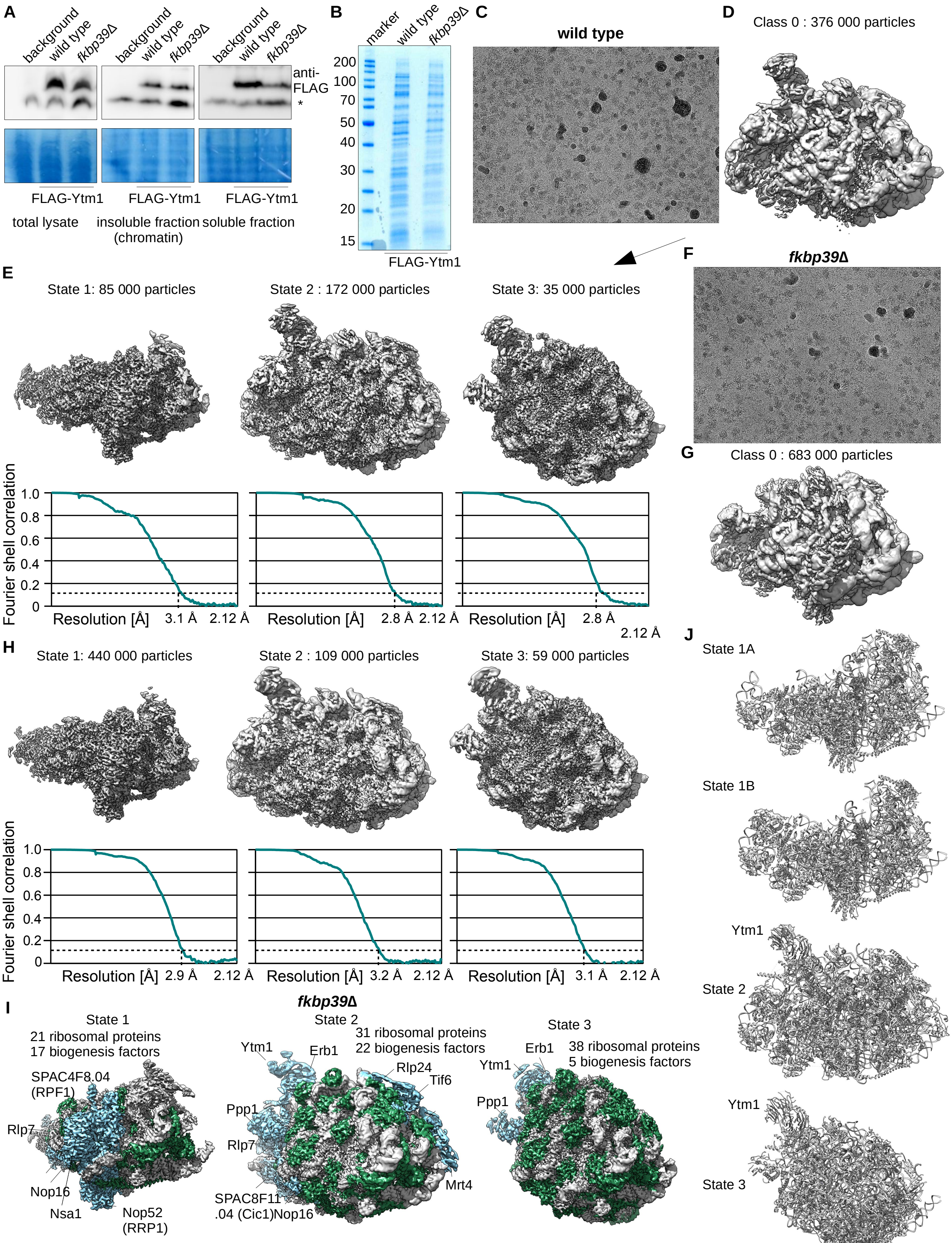

Suppl. Figure 6

Figure S6: Cryo-EM analysis of Ytm1 interacting nascent 60S subunits in wild type and *fkbp39Δ* cells, Related to Figure 5.

A, Western blot showing Ytm1 expression levels in wild type and *fkbp39Δ* cells. Cells expressing FLAG-Ytm1 were lysed and the total, insoluble (chromatin) and soluble fractions were analyzed by anti-FLAG western blot. The amido black stained membrane is showed as loading control. This is a representative image from three independent experiments.

B, SDS gel from the FLAG-Ytm1 purification in wild type and *fkbp39Δ* cells used for structural analysis by cryo-EM.

C, Representative cryo-EM micrograph of Ytm1 interacting particles from wild type cells. Data were collected with Titan Krios electron microscope at 300 keV.

D, Initial map of Ytm1 interacting particles from wild type cells generated from the entire dataset comprising 376 000 particles. The dataset was further extensively classified.

E, Cryo-EM maps of nascent 60S subunits interacting with Ytm1 in wild type cells. Maps are filtered to the corresponding local resolution. The fourier shell correlation (FSC) curve showing the resolution of the map is shown below.

F, Representative cryo-EM micrograph of Ytm1 interacting particles from *fkbp39Δ* cells. Data were collected with Titan Krios electron microscope at 300 keV.

G, Initial map of Ytm1 interacting particles from *fkbp39Δ* cells generated from the entire dataset comprising 683 000 particles. The dataset was further extensively classified.

H, Cryo-EM maps of nascent 60S subunits interacting with Ytm1 in *fkbp39Δ* cells. Maps are filtered to the corresponding local resolution. The fourier shell correlation (FSC) curve showing the resolution of the map is shown below.

I, Cryo-EM maps showing the 3 major states of nascent 60S subunits associated with the biogenesis factor Ytm1 in *fkbp39Δ* cells. Ribosomal RNA is shown in gray, ribosomal proteins in green and ribosome biogenesis factors are colored in blue. All maps are filtered to the corresponding local resolution.

J, Models for different states of Ytm1-containing nascent 60S.

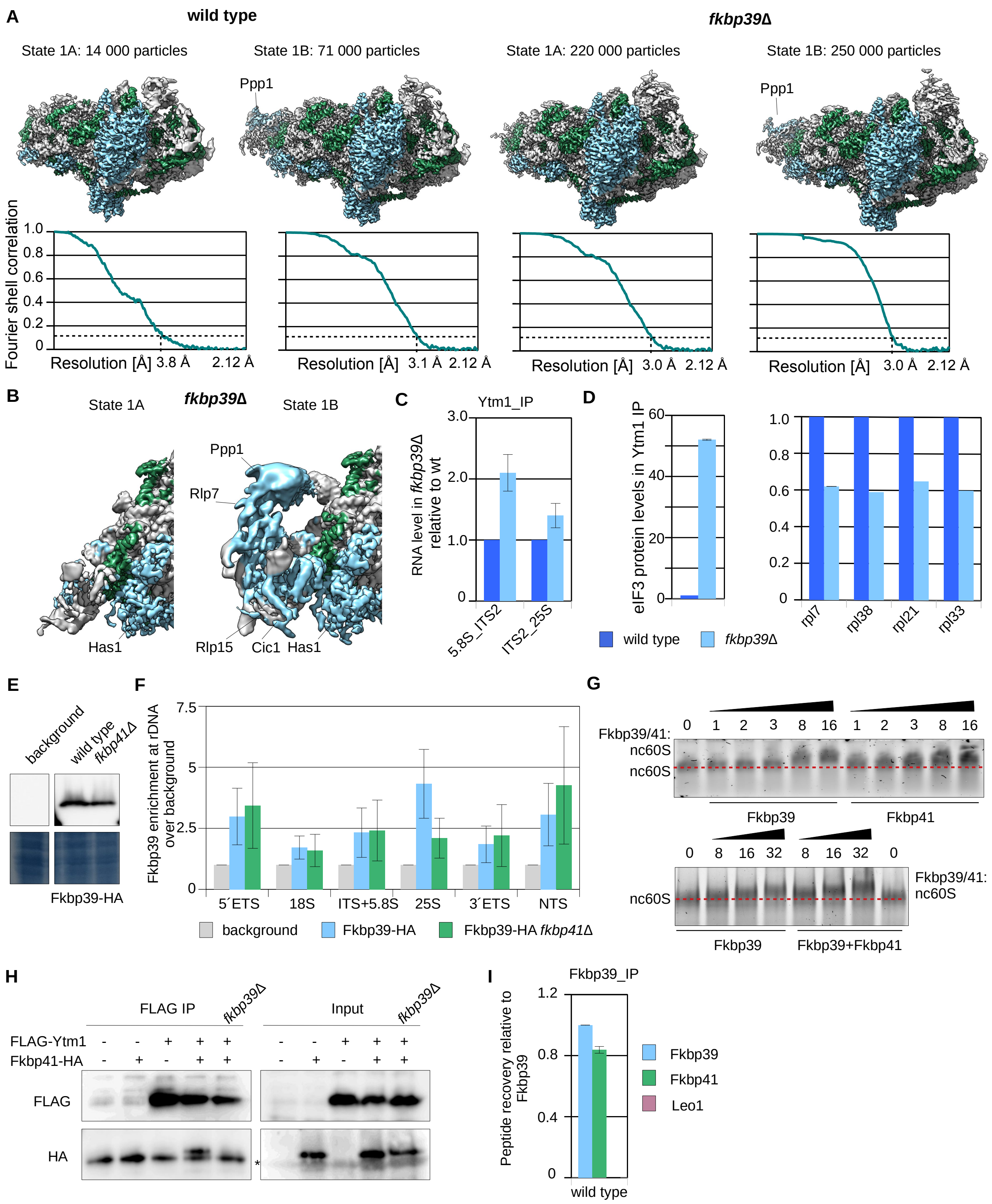

Suppl. Figure 7

Figure S7: Fkbp39 localization to 25S rDNA is required for nascent 60S binding, Related to Figure 6.

A, State 1 was further classified into two sub-states, 1A and 1B. Ribosomal RNA is shown in gray, ribosomal proteins in green and ribosome biogenesis factors are colored in blue. All maps are filtered to the corresponding local resolution. The fourier shell correlation (FSC) curve showing the resolution of the map is shown on the right.

B, Zoom in of states 1A and 1B in *fkbp39Δ* cells. In state 1B Ppp1 and Rlp7 are associated with nascent 60S subunits, but not in state 1A.

C, qPCR quantification of ITS2 splice sites in Ytm1-pulldown from wild type and *fkbp39Δ* cells.

D, Quantification of mass spectrometry data of Ytm1-pulldown from wild type and *fkbp39Δ* cells showing that Ytm1-containing nascent 60S have reduced levels of several ribosomal proteins in *fkbp39Δ* cells. The data also reveal that nascent 60S interact with eIF3 in *fkbp39Δ* cells. Peptide counts of the indicated proteins were normalized to total spectral counts and plotted relative to peptide counts in wild type cells. Quantification is from 2 independent mass spectrometry experiments, error bars represent standard deviation. A complete list of the mass spectrometry results is available in supplementary materials.

E, Western blot showing Fkbp39 expression levels in wild type and fkbp41Δ cells. Cells expressing Fkbp39-HA were lysed and the soluble fraction was analyzed by anti-HA western blot. The amido black stained membrane is shown as loading control. This is a representative image from four independent experiments.

F, Quantification of the reads mapping over rDNA from Fkbp39 ChIP-seq in wild type and *fkbp41Δ* cells. Reads were normalized to background regions and plotted relative to background. Quantification is the average of two independent ChIP-seq experiments and error bars represent the standard error.

G, *In vitro* binding assay showing Fkbp39, Fkbp41 and Fkbp39/41 binding to nascent 60S subunits. The relative molar ratio of Fkbp39 and Fkbp41 in respect to nascent 60S subunits is indicated. The formation of the complexes was visualized on 0.8% TBE agarose gel stained with SYBR gold. The red dash line represents the migration line of nascent 60S subunits. This is a representative experiment of three independent assays.

H, Co-immunoprecipitation experiment between FLAG-Ytm1 and Fkbp41-HA in wild type and *fkbp39Δ* cells. Immunoprecipitates and inputs were analyzed by anti-FLAG and anti-HA western blot. The asterisk represents antibody non specific signal. This is a representative experiment of two independent replicates.

I, Mass spectrometry data of Fkbp39 immunoprecipitation from wild type cells showing that Fkbp39 does not interact with the transcription machinery. Peptide counts of the indicated proteins were normalized to total spectral counts and plotted relative to Fkbp39 peptide counts. Quantification is from

2 independent mass spectrometry experiments, error bars represent standard deviation. A complete list of the mass spectrometry results is available in supplementary materials.

Table S1. Mass spectrometry data of Fkbp39 immunoprecipitation, Related to Figure 1.

Top hits are involved in ribosome biogenesis and were detected in all experiments. Total peptides count and number of unique peptides per protein (in brackets) are shown for control samples and the endogenously tagged strains. A complete list of the mass spectrometry results is available in Data S1.

	No tag	Fkbp39-FLAG-6His	No tag (1)	Fkbp39-FLAG-6His (1)	No tag (2)	Fkbp39-FLAG-6His (2)
Fkbp39	0 (0)	355 (22)	3 (1)	281 (19)	12(6)	597 (34)
Fkbp41	0 (0)	303 (23)	0 (0)	231 (20)	4(2)	232 (26)
Nog1	0 (0)	126 (29)	0 (0)	116 (27)	0 (0)	31 (23)
Ppp1	0 (0)	87 (21)	0 (0)	73 (20)	0 (0)	14 (11)
Nop2	0 (0)	79 (21)	0 (0)	70 (21)	0 (0)	24 (13)
Erb1	0 (0)	60 (23)	0 (0)	60 (17)	0 (0)	5 (5)
Grn1	0 (0)	60 (20)	0 (0)	54 (18)	0 (0)	4 (4)
Ytm1	0 (0)	57 (17)	0 (0)	40 (13)	0 (0)	9 (9)
SPAC8F11.04	0 (0)	53 (18)	0 (0)	52 (14)	0 (0)	5 (4)
Rpf2	0 (0)	53 (11)	0 (0)	54 (11)	0 (0)	13 (10)
Rpl20a	0 (0)	47 (11)	0 (0)	42 (8)	0 (0)	84 (22)
Has 1	0 (0)	46 (15)	0 (0)	53 (20)	0 (0)	11 (7)
Brx1	0 (0)	45 (13)	0 (0)	23 (7)	0 (0)	5 (5)
Nsa2	0 (0)	45 (11)	0 (0)	28 (10)	0 (0)	12 (10)

Table S2. Ribosomal proteins, biogenesis factors and nucleic acids present in the Cryo-EM structures of nascent 60S subunits, Related to Figure 2.

	State 1	State 2	State 3	State 4
**Ribosomal proteins**				
Rpl13	+	+	+	+
Rpl14	+	+	+	+
Rpl15	+	+	+	+
Rpl18	+	+	+	+
Rpl19		+	+	+
Rpl20	+	+	+	+
Rpl21	+	+	+	+
Rpl22			+	+
Rpl27			+	+
Rpl28		+	+	+
Rpl30			+	+
Rpl31	+	+	+	+
Rpl32-1	+	+	+	+
Rpl33	+	+	+	+
Rpl34			+	+
Rpl36	+	+	+	+
Rpl37-2	+	+	+	+
Rpl38-1			+	+
Rpl39				+
Rpl6	+	+	+	+
Rpl8	+	+	+	+
Rpl16	+	+	+	+
Rpl23-1	+	+	+	+
Rpl25a	+		+	+
Rpl26	+	+	+	+
Rpl35	+	+	+	+
Rpl3	+	+	+	+
Rpl4	+	+	+	+
Rpl9-1	+	+	+	+
Rpl7	+	+	+	+
Rpl17	+	+	+	+
**Biogenesis factors**				
Brx1	+		+	

SPAC8F11.04 (CIC1)	+		+	
Erb1	+		+	
Has1	+		+	
Mrt4	+	+	+	+
Nog1	+	+	+	+
SPCC1827.05c (NOP15)	+		+	
Ppp1 (NOP7)	+		+	
Nsa2	+	+	+	+
Grn1 (NUG1)		+		+
Rlp7	+		+	
Tif6	+	+	+	+
SPBC32H8.05 (YBL028C)	+	+		+
Ytm1			+	
Rlp24	+	+	+	+
Nop16	+		+	
Spb1		+		+
Ebp2	+			
SPAC4F8.04 (RPF1)	+			
Mak16	+	+	+	+
Nop52 (RRP1)	+			
Nsa1	+			
**RNA**				
5.8s	+	+	+	+
ITS2	+		+	
25s	+	+	+	+

Table S3. Mass spectrometry data of Nop1 immunoprecipitation, Related to Figure 3.

Top hits are involved in ribosome biogenesis and were detected in both replicates. Total peptides count and number of unique peptides per protein (in brackets) are shown for control samples and the endogenously tagged strains. A complete list of the mass spectrometry results is available in Data S1.

	No tag	FLAG-Nop1	No tag (1)	FLAG-Nop1 (1)
Nop1	0 (0)	194 (17)	0 (0)	139 (15)
Nop56	0 (0)	127 (21)	0 (0)	88 (18)
Dip2	0 (0)	104 (27)	0 (0)	52 (15)
Utp21	0 (0)	95 (24)	0 (0)	39 (14)
Utp13	0 (0)	89 (24)	0 (0)	43 (12)
Utp8	0 (0)	88 (21)	0 (0)	33 (11)
Nop58	0 (0)	81 (17)	0 (0)	50 (13)
Pwp2 (UTP1)	0 (0)	78 (24)	0 (0)	21 (12)
Utp4	0 (0)	76 (17)	0 (0)	47 (13)
Nan1 (UTP17)	0 (0)	75 (19)	0 (0)	31 (12)
Utp10	0 (0)	68 (27)	0 (0)	16 (5)
Utp7	0 (0)	64 (12)	0 (0)	30 (9)
Utp18	0 (0)	62 (13)	0 (0)	17 (5)
Sof1	0 (0)	51 (11)	0 (0)	14 (5)

Table S4. Ribosomal proteins, biogenesis factors and nucleic acids present in the Cryo-EM structures of nascent 60S subunits associated with the biogenesis factor Ytm1 in wild type cells, Related to Figure 5.

	State 1	State 2	State 3
**Ribosomal proteins**			
Rpl13	+	+	+
Rpl14	+	+	+
Rpl15	+	+	+
Rpl18	+	+	+
Rpl19-1		+	+
Rpl20	+	+	+
Rpl21	+	+	+
Rpl22		+	+
Rpl27-1		+	+
Rpl30		+	+
Rpl31		+	+
Rpl32-1	+	+	+
Rpl33	+	+	+
Rpl34		+	+
Rpl36	+	+	+
Rpl37-2	+	+	+
Rpl38-1		+	+
Rpl6	+	+	+
Rpl8	+	+	+
Rpl16	+	+	+
Rpl23-1	+	+	+
Rpl25a		+	+
Rpl26	+	+	+
Rpl35	+	+	+
Rpl3	+	+	+
Rpl4	+	+	+
Rpl9-1	+	+	+
Rpl7	+	+	+
Rpl17	+	+	+
Rpl5			+
Rpl10			+
Rpl11			+
Rpl28		+	+

Rpl39		+	+
Rpl42			+
Rpl43			+
Rpl2			+
Rpl29			+
**Biogenesis factors**			
Brx1	+	+	
SPAC8F11.04 (CIC1)	+	+	
Erb1	+	+	+
Has1	+	+	
Mrt4		+	
Nog1	+	+	
SPCC1827.05c (NOP15)	+	+	
Ppp1 (NOP7)	+	+	+
Nsa2	+	+	
Grn1 (NUG1)		+	
Rlp7	+	+	
Tif6	+	+	
SPBC32H8.05 (YBL028C)		+	
Ytm1		+	+
Rlp24	+	+	+
Nop16	+	+	
Spb1		+	
Ebp2	+	+	
SPAC4F8.04 (RPF1)	+	+	
Mak16	+	+	+
Nop52 (RRP1)	+		
Nsa1	+		
Nop2		+	
Noc3		+	
Nip7		+	
Noc2		+	
**RNA**			
5.8s	+	+	+
ITS2	+	+	
5 s			+
25s	+	+	+

Table S5. Mass spectrometry data of Fkbp39 immunoprecipitation from wild type and *fkbp41Δ* cells, Related to Figure 6.

Top hits are involved in ribosome biogenesis. Total peptides count and number of unique peptides per protein (in brackets) are shown for control samples and the endogenously tagged strains. Proteins marked by ** are above the set threshold for both *fkbp41Δ* samples, whereas the ones marked by * only for one. A complete list of the mass spectrometry results is available in Data S1.

	No tag	Fkbp39-FLAG-6His	Fkbp39-FLAG-6His fkbp41Δ (1)	Fkbp39-FLAG-6His fkbp41Δ (2)
Fkbp39**	5 (2)	236 (18)	206 (18)	195 (20)
Fkbp41	0 (0)	267 (24)	0 (0)	0 (0)
Nog1	10 (8)	159 (34)	25 (12)	26 (13)
Ppp1	2 (1)	126 (22)	8 (5)	4 (3)
Nop2	2 (1)	102 (18)	4 (3)	7 (4)
Erb1*	0 (0)	136 (28)	0 (0)	6 (4)
Grn1**	0 (0)	86 (24)	7 (5)	6 (4)
Ytm1**	0 (0)	99 (18)	7 (4)	8 (4)
SPAC8F11.04**	0 (0)	102 (23)	6 (3)	7 (3)
Rpf2*	0 (0)	68 (12)	4 (3)	5 (3)
Rlp7**	0 (0)	71 (13)	28 (10)	30 (10)
Has1*	0 (0)	106 (21)	3 (2)	5 (3)
Wdr55**	0 (0)	25 (8)	5 (2)	5 (2)
Nop56**	0 (0)	11 (4)	8 (3)	10 (5)

Table S6. **Mass spectrometry data of Fkbp41 immunoprecipitation from wild type and *fkbp39Δ* cells, Related to Figure 6.**

Top hits are involved in ribosome biogenesis and transcription. Total peptides count and number of unique peptides per protein (in brackets) are shown for control samples and the endogenously tagged strains. Proteins marked by ** are above the set threshold for all samples, whereas the ones marked by * only for some. A complete list of the mass spectrometry results is available in Data S1.

	No tag	Fkbp41-FLAG-6His	Fkbp41-FLAG-6His fkbp39Δ	No tag (2)	Fkbp41-FLAG-6His (2)	Fkbp41-FLAG-6His fkbp39Δ (2)
Fkbp41**	0 (0)	152 (14)	75 (11)	0 (0)	78 (9)	25 (3)
Fkbp39*	0 (0)	219 (9)	0 (0)	2 (2)	107 (7)	0 (0)
Nog1*	4 (3)	47 (10)	16 (6)	0 (0)	4 (2)	0 (0)
Ppp1*	3 (2)	19 (6)	11 (4)	0 (0)	4 (2)	0 (0)
Nsa1**	0 (0)	16 (3)	7 (1)	0 (0)	17 (3)	7 (4)
Ytm1*	2 (2)	33 (8)	17 (4)	0 (0)	2 (1)	0 (0)
Grn1*	0 (0)	6 (4)	0 (0)	0 (0)	10 (3)	0 (0)
Ebp2*	2 (1)	5 (2)	3 (1)	0 (0)	10 (1)	4 (1)
Has1*	0 (0)	16 (4)	9 (4)	0 (0)	6 (2)	0 (0)
Leo1*	0 (0)	0 (0)	13 (2)	0 (0)	0 (0)	4 (1)
Mas5*	0 (0)	25 (2)	12 (2)	0 (0)	0 (0)	18 (2)

Table S7. *S. pombe* strains used in this study, Related to STAR methods.

63	h+ *otrR*(SphI)*::ura4 ura4 DS/E leu1-32 ade6-M210*	SPY137
65	h+ *otr1R*(SphI)*::ura4+ ura4-DS/E leu1-32 ade6-M210 natMx6::*3xFLAG-*ago1*	
860	h+ *leu1-32 ade6-M216 ura4-D18 his3 fkbp39*-HA*::kanMX6*	SPY79
1164	h+ *otrR*(SphI)*::ura4 ura4 DS/E leu1-32 ade6-M210 fkbp41*-3HA*::kanMX6*	
1315	h- *leu1-32 fkbp39*-1xFLAG6His::*hphMX6*	
1323	h- *leu1-32 1-768fkbp39*-1xFLAG6His::*hphMX6*	
1331	*leu1-32 fkbp41*-3HA::*kanMX6 fkbp39*-1xFLAG6His::*hphMX6*	
1334	*leu1-32 fkbp41*-3HA::*kanMX6 1-768fkbp39*-1xFLAG6His::*hphMX6*	
1340	*leu1-32 fkbp41*-3HA::*kanMX6 fkbp39*Δ::*natMX6*	
776	h+ *otr1R*(SphI)::*ura4+ ura4-DS/E leu1-32 ade6-M210*<br>*natMx6*::3xFLAG-*ago1 fkbp39*Δ::*kanMX6*	
986	h+ *otrR*(SphI)*::ura4 ura4 DS/E leu1-32 ade6-M210*<br>*fkbp39*-2XFLAG6His::*hphMx6*	
1172	h+ *otrR*(SphI)*::ura4 ura4 DS/E leu1-32 ade6-M210 fkbp41*Δ::*hphMx6*	
1173	h+ *otrR*(SphI)*::ura4 ura4 DS/E leu1-32 ade6-M210 fkbp41*Δ::*hphMx6*<br>*natMx6*::3xFLAG-*ago1 fkbp39*Δ::*kanMX6*	
1314	h+ *otrR*(SphI)::*ura4 ura4 DS/E leu1-32 ade6-M210*<br>*natMx6*::1xFLAG-*ytm1*	
1318	h- *leu1-32 natMx6*::1xFLAG-*ytm1 fkbp39*Δ::*kanMx6*	
1335	h+ *leu1-32 natMx6*::1xFLAG-*ytm1 fkbp39*-HA::*kanMX6*	
1338	*leu1-32 natMx6*::1xFLAG-*ytm1 1-768fkbp39*-3xHA::*kanMX6*	
1341	h+ *otrR*(SphI)*::ura4 ura4 DS/E leu1-32 ade6-M210*<br>*hphMx6*::1xFLAG-*ytm1*	
1342	h+ *otrR*(SphI)::*ura4 ura4 DS/E leu1-32 ade6-M210*<br>*hphMx6*::1xFLAG-*ytm1 fkbp41*-3HA::*kanMX6*	
1343	*leu1-32 hphMx6*::1xFLAG-*ytm1 fkbp41*-3HA::*kanMX6 fkbp39*Δ::*natMx6*	
1435	h+ *leu1-32 natMx6*::1xFLAG-*ytm1 fkbp39*-HA::*kanMX6 fkbp41*Δ::*hphMx6*	
1436	*leu1-32 natMx6*::1xFLAG-*ytm1 1-768fkbp39*-3xHA::*kanMX6 fkbp41*Δ::*hphMx6*	
1447	h+ *otrR*(SphI)*::ura4 ura4 DS/E leu1-32 ade6-M210 natMx6*::1xFLAG-*nop1*	
1469	*leu1-32 fkbp39*-1xFLAG6His::*hphMX6 fkbp41*Δ::*natMx6*	
1349	*otrR*(SphI)::*ura4 ura4 DS/E leu1-32 ade6-M210*<br>*natMx6*::1xFLAG-*ytm1 fkbp41*Δ::*hphMx6*	
1346	*leu1-32 natMx6*::1xFLAG-*ytm1 fkbp39*Δ::*kanMx6 fkbp41*Δ::*hphMx6*	
1473	h+ *otrR*(SphI)*::ura4 ura4 DS/E leu1-32 ade6-M210*<br>*fkbp41*-3xFLAG6His::*hphMX6*	
1474	*leu1-32 fkbp41*-3xFLAG6His::*hphMX6 fkbp39*Δ::*kanMX6*	

Table S8. Plasmids used in this study, Related to STAR methods.

p298	pET3a 601 nucleosome sequence
p822	pET Duet 6His-Sumo-Fkbp39
p1300	pET Duet 6His-Sumo-Fkbp39_cys
p85	pFA6a 3XHA::*kanMx6*
p780	pFA6a 3XFLAG6His::*hphMx6*
p1224	pFA6a *1-768fkbp39*-3XHA::*kanMx6*
p1225	pFA6a *1-768fkbp39*-3XFLAG6His::*hphMx6*
p1196	pFA6a *natMx6::ytm1*promoter-1XFLAG
p1322	pET Duet 6His-Sumo-Fkbp39_1-198
p1323	pET Duet 6His-Sumo-Fkbp39_199-361
p1331	pET Duet 6His-Sumo-Fkbp39_1-256
p1330	pFA6a *natMx6::nop1*promoter-1XFLAG
p1333	pET Duet 6His-Sumo-Fkbp41
p1335	pET Duet 6His-Sumo-Fkbp39F301CW314CY337K
SJ224	pET Duet 6His-Sumo-Fkbp41 cys

Table S9. Oligos used in this study, Related to STAR methods.

113C	25S rDNA	TTTTCTCCTTCTCGGGGATT	qPCR
113D	25S rDNA	AACACCACTTTCTGGCCATC	qPCR
252F	fkbp39	ACTTAATGGAATACACAACGGTTTATGTATCAAAATTTTTACATC CTCTACCACCTCACCTCTTAGGCAAGAATAAGTCCGGATCCCC GGGTTAATTAA	cloning
252R	fkbp39	AGAGTTAATAGAATACATAGTTTAATCCGCGATCTTCTTAAACAC GGGAGTTAAAGTATGTTCATCTTCAACATGAAAATGAATTCGAG CTCGTTTAAAC	cloning
531F	fkbp39	TGGCTTACGGCAACCAGAGCATTCCAGGAATTCCCAAGAATTC TACCTTAGTTTTTGAAGTCAAGCTTGTTCGCGTTCACCGGATCC CCGGGTTAATTAA	cloning
531R	fkbp39	AGAGTTAATAGAATACATAGTTTAATCCGCGATCTTCTTAAACAC GGGAGTTAAAGTATGTTCATCTTCAACATGAAAATGAATTCGAG CTCGTTTAAAC	cloning
750F	fkbp39	TCCTTGGTCGTGGTGAG	cloning
750R	fkbp39	TAAAAGCACAGGGTTTACCT	cloning
751F	fkbp39	CGGCGTTGCTGGAATGC	cloning
751R	fkbp39	ACGTCACACCCGCGAATA	cloning
752F	fkbp39	TGGCTAA AGGCAACCAGAG	cloning
752R	fkbp39	TGGGAGCAGGAATTGTAATC	cloning
753F	fkbp39	GTACCGAGCTCTAAA AGG	cloning
753R	fkbp39	TTATTTTAAAGTACGAGTCTTGG	cloning
1287F1	fkbp39	ACTTAATGGAATACACAACGGTTTATGTATCAAAATTTTTACATC CTCTACCACCTCACCTCTTAGGCAAGAATAAGTCATGTCTCTTC CAATTGCTGT	cloning
1288F	fkbp39	ATTGGAAGTGGATAACATGTCTCTTCCAATTGCTGT	cloning
1288R	fkbp39	AATGGATTGGAAGTACGTGAACGCGAACAAGCT	cloning
1289F		TACTTCCAATCCATTGTACCCATACGATGTTCCT	cloning
1289R		TTATCCACTTCCAATGTTAATTAACCCGGGGATC	cloning
1290F		TACCCATACGATGTTCCT	cloning
1290R		GTGAACGCGAACAAGCT	cloning
1299F	fkbp39	CTACTGGTCCCGCTGCTAAGAAAGAAAAGCAACAAGCTTCTTC TAATGCACCTTCTAGTCCCAAGACTCGTACTTTAAAAATCCCCG GGTTAATTAACAT	cloning
1308R	fkbp39	TTTTAAAGTACGAGTCTTGG	cloning
1376F	fkbp39	GCTAACTCGAGTCTGGTAAAGA	cloning
1376R	fkbp39	AGTGAACGCGAACAAGCT	cloning
533F	fkbp39	CACGGATCCATGTCTCTTCCAATTGCTGTTTATAGT	cloning
533R	fkbp39	CACCTCGAGTTAGTGAACGCGAACAAGCTT	cloning
987F	fkbp41	GATATACCAGTGGTGAATTTCTGTGATTCCCATCGTCTATTGCGT ATCTTTTTGGATTTTATTTATTCTAAATAAACAAACGGATCCCCG GGTTAATTAA	cloning

987R	fkbp41	TAATATTCAAAAAATTTGCTATTCCGTAATTTCCAGACCCAACTA AACCATAATTTTTTTATGAACCAAACTACACAAAAGAATTCGAGC TCGTTTAAAC	cloning
988F	fkbp41	TGGCCTATGGCTCTAAACGTCTTCCAGGTATCCCAGCCAATTCA GATTTGGTTTTTGACGTAAAGCTTTTAGCGGTTAAT - CGGATCCCCGGGTTAATTAA	cloning
1298F	ytm1	ATTCAGCCAAAAGTCATTATCCTCATTTTTTTGGTACTGGAAATT TTGATCTTTTCGACACTCCTTCCACCAACAACAGAGAGCTCGT TTAAACTGGA T	cloning
1298R	ytm1	GGCGTGTCACCAACAGCTAAATCCTCATTTCTAGTAGTAAAACG GACTTGAACTTGGCCTGAAGGAGCGCTTTGGGCATCGCCCTT GTCATCGTC	cloning
1282F	ytm1	ATTGGAAGTGGATAACATCTCCCTTAAAGCAACT	cloning
1282R	ytm1	AATGGATTGGAAGTACTCTGTTGTTGGTGGAAG	cloning
1284F	ytm1	ATGGATTATAAAGATGACGATG	cloning
1284R	ytm1	TCTGTTGTTGGTGGAAG	cloning
797F		TATCCGACTGGCACCG	Nucleosomal DNA
797R		GAGTTCATCCCTTATGTGAT	Nucleosomal DNA
423F		GCACAGGATGTATATATCTG	Nucleosomal DNA
423R		CTGGAGAATCCCGGT	Nucleosomal DNA
1371		GATTTGAGGTCAA-Atto488	probe
1372		ACGTTGCCGTGTTGATT-Atto488	probe
1373		TTTCTGGTGTCTGAT-Atto488	probe
1376F		GCTAACTCGAGTCTGGTAAAGA	cloning
1376R		AGTGAACGCGAACAAGCT	cloning
1397F		ATTGGAAGTGGATAACTGTCTTCAGATGACGAAG	cloning
1397R		AATGGATTGGAAGTACTATTTTTTTTTGTGGTAGGTAAT	cloning
1399F	Nop1	TGTTCGTAGTCACACCAACTGTAATCCTCTCTGCTATCCTAAAA ATTTTACAGACCCATTACCTACCACAAAAAAAAATA GAATTCGAGCTCGTTTAAAC	cloning
1399R	Nop1	AAACCACCACGGCCACCATTGAAACCACCACGGCCTCCTCTA GAGCCACCTCGTCCACCTCTTGAACCTGGTGTATATGC cttatttagaagtggcgcgcc	cloning
R_199	Fkbp39	AGCAGCCTCTTCTTGCT	cloning
F_stop	Fkbp39	TAACTCGAGTCTGGTAAAGAA AC	cloning
R_256	Fkbp39	TTTTAAAGTACGAGTCTTGGGA	cloning
R_sumo	Fkbp39	CATGGATCCGGGCCC	cloning
F_199	Fkbp39	TCTCCTAAGAAAAACAATACCAAG	cloning
1405F	Fkbp41	ATTGGAAGTGGATAACATG AGT AAG GAA GAG ACC	cloning
1405R	Fkbp41	AATGGATTGGAAGTACTTAATTAACCGCTAAAAGCT	cloning

1406F	Fkbp41	TACTTCCAATCCATTGCTC GAG TCT GGT AAA GAA	cloning
1406R	Fkbp41	TTATCCACTTCCAATG GGATCCGGGCCC	cloning
1407F	Fkbp41	ATG AGT AAG GAA GAG ACC	cloning
1407R	Fkbp41	GGATCCGGGCCC	cloning
113C	25S rDNA	TTTTCTCCTTCTCGGGGATT	qPCR
113D	25S rDNA	AACACCACTTTCTGGCCATC	qPCR
SJ135F	ITS2_25S rDNA	TGAACGTAATAGGTTTTACCACTTTGT	qPCR
SJ135R	ITS2_25S rDNA	TGAGCTTTTCCCGCTTCACT	qPCR
SJ143F	25S rDNA_3'ETS	CGAAGCAGAATTCGGTAAGC	qPCR
SJ143R	25S rDNA_3'ETS	GCCGCAAATACCAATGATCT	qPCR
SJ168F	5'ETS	TCCTGATTTTGTTGGGAAGAA	qPCR
SJ168R	5'ETS	AGAGGTGAGACAAAAAGCAGA	qPCR
SJ169F	NTS1	CGCGGTGCATTGGAAAGAAA	qPCR
SJ169R	NTS1	TCCCCTTCTTCTCGTTCAACC	qPCR
SJ133F	5.8S rDNA_ITS2	TGAAGAACGCAGCGAAATGC	qPCR
SJ133R	5.8S rDNA_ITS2	CCTTTTGTTTTCACCAATCGATTTCA	qPCR
SJ134F	ITS2	TCGATTGGTGAAAACAAAAGG	qPCR
SJ134R	ITS2	TTCTATCAAACAAAGTGGTAAAACC	qPCR
SJ137F	TDH1	CCAAGCCTACCAACTACGA	qPCR
SJ137R	TDH1	AGAGACGAGCTTGACGAA	qPCR
SJ140F	Fkbp41	TAAGTACTTCCAATCCATTGC	cloning
SJ140R	Fkbp41	GCAATTAACCGCTAAAAGCTTTACG	cloning

Table S10: Sequencing data, Related to STAR methods.

**ChIP sequencing**	**RNA sequencing**
wild type (63)_Fkbp ChIP seq	wild type (1314)_total RNA seq
Fkbp39-HA (860)_Fkbp ChIP seq	*fkbp39*Δ (1318)_total RNA seq
Fkbp39-HA *fkbp41*Δ (1435)_Fkbp ChIP seq	*fkbp41*Δ (1349)_total RNA seq
	*fkbp39*Δ*fkbp41*Δ (1346)_total RNA seq
Fkbp41-3xHA (1164)_Fkbp ChIP seq	
wild type (63)_Ytm1 ChIP seq	wild type (63)_nascent RNA seq
1xFLAG-Ytm1 (1314)_Ytm1 ChIP seq	*fkbp39*Δ (776)_nascent RNA seq
1xFLAG-Ytm1 *fkbp39*Δ (1318)_Ytm1 ChIP seq	
1xFLAG-Ytm1 *fkbp41*Δ (1349)_Ytm1 ChIP seq	
1xFLAG-Ytm1 *fkbp39*Δ *fkbp41*Δ (1346)_Ytm1 ChIP seq	
1xFLAG-Ytm1 *fkbp41*Δ (1435)_Ytm1 ChIP seq	
